# Predicting Parkinson disease related genes based on PyFeat and gradient boosted decision tree

**DOI:** 10.1038/s41598-022-14127-8

**Published:** 2022-06-15

**Authors:** Marwa Helmy, Eman Eldaydamony, Nagham Mekky, Mohammed Elmogy, Hassan Soliman

**Affiliations:** Information Technology Department, Faculty of Computers and Information, Mansoura University, Mansoura, 35516 Egypt

**Keywords:** Computational biology and bioinformatics, Biomarkers, Engineering, Mathematics and computing

## Abstract

Identifying genes related to Parkinson’s disease (PD) is an active research topic in biomedical analysis, which plays a critical role in diagnosis and treatment. Recently, many studies have proposed different techniques for predicting disease-related genes. However, a few of these techniques are designed or developed for PD gene prediction. Most of these PD techniques are developed to identify only protein genes and discard long noncoding (lncRNA) genes, which play an essential role in biological processes and the transformation and development of diseases. This paper proposes a novel prediction system to identify protein and lncRNA genes related to PD that can aid in an early diagnosis. First, we preprocessed the genes into DNA FASTA sequences from the University of California Santa Cruz (UCSC) genome browser and removed the redundancies. Second, we extracted some significant features of DNA FASTA sequences using the PyFeat method with the AdaBoost as feature selection. These selected features achieved promising results compared with extracted features from some state-of-the-art feature extraction techniques. Finally, the features were fed to the gradient-boosted decision tree (GBDT) to diagnose different tested cases. Seven performance metrics were used to evaluate the performance of the proposed system. The proposed system achieved an average accuracy of 78.6%, the area under the curve equals 84.5%, the area under precision-recall (AUPR) equals 85.3%, F1-score equals 78.3%, Matthews correlation coefficient (MCC) equals 0.575, sensitivity (SEN) equals 77.1%, and specificity (SPC) equals 80.2%. The experiments demonstrate promising results compared with other systems. The predicted top-rank protein and lncRNA genes are verified based on a literature review.

## Introduction

Parkinson’s disease (PD) is a common neurodegenerative disease characterized by the loss of dopaminergic neurons in an area of the brain known as the substantia nigra^1^. This loss in dopaminergic neurons causes unexplained nerve dysfunction, which leads to motor and nonmotor disturbances^2^. PD affects an estimated 7–13 million people worldwide^3^. PD is determined to be rare before the age of 50 years, but it becomes more common as people get older. It affects more than $1\%$ of the people above the age of 60 years and approximately $4\%$ above 80 years. Therefore, PD is considered the most common movement disorder and the second most common neurodegenerative disease after Alzheimer’s disease (AD)^4^. There are four essential signs related to PD: tremor, rigidity, bradykinesia, and postinstability^5^. However, the cause of PD remains unclear. Furthermore, the disease progresses at a different pace in different people. Hence, the disease course varies depending on the patient’s age, and the rate of progression differs across the population^2,6^. PD’s progression and the degree of symptoms create several socioeconomic challenges, affecting PD patients, their caregivers, and the healthcare system^3,4^.

Because of the complexities of PD, there is no single suitable gold standard test to diagnose PD, track its progression, predict risk factors, or assess the PD severity. As a result, there has been an ongoing search for suitable PD biomarkers over the last decade^2,7^. The biomarker is characterized as a noticeable feature that is capable of detecting unusual biological processes^8^. So that, the discovery and validation of PD biomarkers are critical for enhanced clinical evaluation and treatment of the disease.

There are four biomarkers to identify PD: clinical, imaging, biochemical, and genetic markers. Clinical biomarkers have been identified as the most commonly used diagnostic measures, which experts use for assessing and diagnosing PD and determining the progression and severity of PD^1,9,10^. Observing motor symptoms, such as tremor, rigidity, bradykinesia, and postinstability, are considered the primary assessment using the Unified Parkinson Disease Rating Scale (UPDRS). However, distinguishing PD from other parkinsonism and movement diseases, such as progressive supranuclear palsy (PSP) and essential tremor (ET), can be difficult with such markers^2^.

In the neuroimaging biomarkers, PD is characterized by the loss and degradation of the dopaminergic neuron. Consequently, neuroimaging techniques for the dopamine system may be good candidates for diagnosis and treatment analysis^8^. Single-photon emission-computed tomography (SPECT) and dopamine transporter (DAT) imaging modalities have been used widely for diagnosing PD and other neurodegenerative disorders. Other imaging techniques, such as transcranial sonography (TCS) and magnetic resonance imaging (MRI), are also used to track and monitor brain changes that can be used to identify the PD’s risk^11^.

Biochemical biomarkers have benefits over other types of biomarkers. This is because it can be discovered in body fluids, such as saliva, serum, cerebrospinal fluid (CSF), blood, and biopsies, making them less expensive to extract. Consequently, the process includes a noninvasive analysis of the molecules and proteins present in the body fluids^2^. On the other hand, there are 5–10% known genes related to PD as genetic biomarkers, according to the national center for biotechnology information (NCBI) website^4^ and based on the clinical picture of PD for patients^12^. However, approximately $90\%$ of PD genes have not yet been identified. Additionally, PD has various signs, which appear in the latter stages of the disease. Therefore, we work on the genetic markers to identify genes for an early PD diagnosis.

Identifying genes related to diseases is considered a challenging task in biological analysis^13,14^. Nevertheless, it provides significant contributions to understanding disease parthenogenesis, medical diagnosis, and drug development^15,16^. Thus, identifying genes related to PD enhances the experience and understanding of this disease, and helps its diagnosis and treatment of the PD^17^. Several existing methods have been designed for predicting disease-related genes. However, a few of these methods are used for PD gene prediction^18–22^. Furthermore, a few PD methods are designed to identify genes that can code as proteins and discard noncoded elements^17,23–25^, such as long noncoding RNAs (lncRNAs) and microRNAs (miRNAs) in PD gene prediction^22^.

Most studies in the biological field show that lncRNAs play a critical role in transforming and developing various diseases. The lncRNA is a transcript of more than 200 nucleotides that cannot be translated into proteins. lncRNAs are essential in many fundamental biological processes, such as post-transcriptional and transcriptional regulation, epigenetic regulation, cell cycle control, cell differentiation and apoptosis, cellular transport, organ or tissue development, chromosome dynamics, and metabolic processes. Therefore, the mutations and dysregulations of lncRNAs would aid in developing various human complex diseases^26^.

Identifying lncRNAs associated with diseases is vital for improving the diagnosis and treatment of the diseases. A long time ago, some studies proposed models for predicting and identifying lncRNAs related to diseases, the Laplacian Regularized Least Squares for LncRNA–Disease Association (LRLSLDA) model is the first computational model for identifying lncRNA–disease associations^27,28^. Therefore, identifying protein and lncRNA genes related to PD enhances its diagnosis and treatment^21,22^.

Our proposed prediction system used the lncRNA genes as another data source besides the protein genes. The use of lncRNAs overcomes the limitation that only protein genes are expressed as the original data. We can identify all genes associated with PD, which can aid in an early diagnosis and treatment. We represent all genes into deoxyribonucleic acid (DNA) FAST-All (FASTA) sequences that contain the most significant information about the genes. Its play an important role in the extracting of essential and distinguishing features of the genes^29^. The main contributions of our proposed prediction system can be summarized in the following points:

- A novel framework is proposed for predicting genes related to PD based on protein and lncRNA genes, which play a critical role in PD development.
- All protein and lncRNA genes are presented as DNA FASTA sequences to obtain local and global significant genes. The FASTA sequences are fed to multiple feature extraction methods to extract the most distinguishing and vital features.
- The PyFeat method is used to achieve this goal. Then, the AdaBoost (AB) technique is used to reduce the dimensionality of the PyFeat features generation and decrease the complexity and computational time.
- The most distinguishing features are fed to the gradient-boosted decision tree (GBDT) technique to diagnose different test cases. Then, various performance metrics are used to evaluate the proposed system. Additionally, we validated our proposed system by comparing it to some current systems. We verified the predicted top-rank protein and lncRNA genes based on the most recent studies from the literature.

For the reader’s convenience, the used abbreviations in this paper are listed in Table 1. The rest of this paper is divided into five sections. Section “Related work” discusses the related work, current weaknesses, and how we overcome these limitations in our proposed system. The materials and methods are introduced in next section. The datasets, hardware specifications, evaluation metrics, and results are introduced in section “Experimental results”. Section “Discussion” discusses our experimental results. Finally, last section represents a conclusion and summary of our future work plans.

**Table 1:** The used abbreviations.

PD	Parkinson’s disease	ACC	Accuracy
lncRNA	Long non coding RNA	PPV	Positive predictive value
DFT	Discrete Fourier Transform	FFT	Fast Fourier Transform
MM	monoMonoKGap	MD	monoDiKGap
MT	monoTriKGap	DM	diMonoKGap
DD	diDiKGap	DT	diTriKGap
TM	triMonoKGap	TD	triDiKGap
A	Adenine	C	Cytosine
G	Guanine	T	Thymine
DT	Decision Tree	NB	Naive Bayes
TP	True positive	RF	Random Forest
FP	False positive	AB	Adaboost
LR	Logistic Regression	GBDT	gradient boosting decision tree
SVM	Support Vector Machine	LDA	Linear Discriminant Analysis
AUPR	Area under precision-recall	AUC	Area Under the Curve
FN	False negative	TN	True negative
SE	Sensitivity	SPC	specificity
TPR	True positive rate	FPR	False negative rate

## Related work

Predicting genes related to a disease is considered an active search topic in the biological field. Many researchers have identified and predicted genes related to these diseases; some of these studies have specialized in PD. Table 2 shows a summary of the current studies. Some studies built models for identifying and predicting diseases-genes, and ignoring lncRNAs related to diseases. For example, Radivojac et al.^18^ presented an approach to predict the disease-related genes based on the protein-protein interaction (PPI) network. First, they presented feature vectors in three ways: disease–protein relationship, protein sequence, and protein function information. Second, they used information gain to rank features, reducing the feature vector dimension to overcome overfitting and computation costs. Finally, in the classification step, they applied the support vector machine (SVM) classifier as a supervised technique with two layers for predicting genes related to the disease.

Zhang et al.^23^ performed frequent gene co-expression analysis to identify genes associated with PD. They used six known genes related to PD as known genes. They used Pearson correlation coefficients (PCC) between any couple of genes inside each dataset to find genes that frequently co-express with these known genes. A set of PD genes were identified. This set of genes was analyzed and showed great importance in neurodegenerative diseases and metabolism. Yang et al.^19^ proposed a novel ensemble-based PU learning method (EPU) to identify genes related to the disease. They used multiple data sources and ensemble machine learning classifiers. First, they built three networks: the PPI, GO similarity, and gene expression similarity networks. They applied weighted K-nearest neighbor (KNN), weighted naïve Bayes (NB), and multiple level SVM classier based on the ensemble weighted gene. Based on ensemble-weighted classifiers, they built the EPU learning to predict disease-related genes.

Peng et al.^30^ built an integrated network containing different nodes and edges. It presented various biomedical data, such as diseases, genes, ontology terms, and their associations. They developed a simplified laplacian normalization supervised random walk (SLNSRW) algorithm, which comprises three steps. First, they used multiple datasets and ontologies to build an integrated network. Second, they built a weighted integrated network using a laplacian normalization. Finally, they applied a supervised random walk (RWR) method to predict disease-related genes based on a weighted integrated network.

Hwang^20^ presented stepwise random forests (SRF) method to select the biological features to identify genes related to the disease. They integrated multiple biological features from the gene characteristics: protein domains, gene ontology, and human protein interactions. They conducted phenotype-gene association and preliminary feature selection. The SRF method comprises two steps. First, the most important features were selected using filter-based methods according to one-dimensional random forest regression. Second, the selected biological features were fed to random forest classification for identifying genes related to the disease.

Tian et al.^31^ developed a random walk with restart on the phenotype-gene bilayer network (RWRB) method to identify disease-related genes. First, they built different gene similarity networks based on various genomic data of genes. Second, the integrated gene similarity network (IGSN) was built based on the technique of similarity network fusion (SNF). Finally, they used EWRB, which merged phenotype network, IGSN, and gene-phenotype network to identify disease-related genes. Peng et al.^17^ identified genes related to PD based on node2vec autoencoder-support vector machine (N2A-SVM) method. They aimed to identify the protein genes related to PD. Their method comprises three steps. First, they represented each gene using the PPI network. Second, they used node2vec to extract the important features of these representations. Third, for dimension reduction of features, they used the auto-encoder method. Finally, they used the SVM classifier to build their training model.

Yang et al.^24^ predicted the disease-related genes using a novel deep neural network model (PDGNet). They combined multiple views of phenotypes and genotypes features. They enhanced the deep neural network parameters and extracted an accurate features vector for each gene and disease with feedback information from training samples. These vectors were used as input layers in their non-linear network for learning multiple features of genes and disease. The appropriate scores between genes and disease were calculated by determining the similarity among their vectors. They used the cross entropy between the relevant scores and the true labels of disease–gene relations to optimize their model as the feedback results.

Joodaki et al.^32^ integrated multiple protein/gene networks to overcome the false positive interaction prediction. They built a heterogeneous network based on gene-gene associations, disease–disease associations, and disease–gene associations. They developed a method, namely random walk with restart on the heterogeneous network method with fuzzy (RWRHN-FF). First, they constructed four gene-gene association networks, and these networks were integrated as a network based on a type-II fuzzy voter scheme. Second, the disease–disease association networks from four sources were linked to the integrated gene-gene network. Finally, they applied the RWRHN-FF method to rank the disease–gene associations using the Apache spark for parallel implementation.

Bi et al.^25^ used data to design a realistic multimodal analysis model from functional magnetic resonance imaging (fMRI) and single nucleotide polymorphisms (SNPs). Their model consisted of three parts. First, they used correlation analysis to build the subject’s fusion. Second, they analyzed the fusion feature using their neural network as a clustering evolutionary random neural network ensemble (CERNNE). Finally, their method combined random neural networks and used the clustering technique for optimizing the ensemble learner. The CERNNE was used to create a multi-task research system, identify PD patients, and predict PD-related genes and brain regions.

On the other hand, some studies are also interested in predicting and identifying lncRNAs related to diseases. For example, Ding et al.^21^ proposed a prediction model for identifying the lncRNA–disease relationship via tripartite graph lncRNA–disease–gene (TPGLDA). Their model consists of four steps. First, they built gene–disease and lncRNA–disease adjacency matrix by combining gene–disease and lncRNA–disease interactions. Second, they estimated the relationship profile for each node, combined this vector into the adjacency matrix to allocate resources, and built a tripartite graph based on lncRNA, disease, and gene. Third, they used the resource allocation process according to a tripartite graph to build the relationship between lncRNA and disease. Finally, for each disease–lncRNA relationship, they calculated the resource score consequently.

Lei et al.^15^ identified genes related to common diseases, including PD. They combined protein genes, lncRNAs, and diseases with building a heterogeneous network. They proposed a network propagation algorithm to be applied to these heterogeneous networks. They employed the information loss model to improve these networks for identifying genes related to the disease. They determined the weights of the similarity networks based on information loss to select the most important relationships using 3-sigma. They used a network propagation algorithm to score genes. The disease–genes association probabilities were represented based on the final score of these genes.

Xuan et al.^22^ proposed a method for identifying lncRNA genes related to the disease. They presented a convolutional neural network (CNN) to predict the lncRNA–disease associations referred to as CNNLDA. Their system determined the similarities and relationships: lncRNAs–diseases, lncRNAs–miRNAs, and miRNA–disease relationships. They combined these similarities and relationships to build the matrix of features based on the biological principles of diseases, lncRNAs, and miRNAs. Thus, their framework was designed to extract both the attention and the global feature representations of disease–lncRNA relationships. The first part of their framework was specialized for feature extraction from the similarities and associations of diseases and lncRNAs. In the second part of their framework, the various weights were assigned to each feature and its types by performing their proposed system to predict lncRNAs related to the disease.

Zhang et al.^33^ identified and predicted the relationships between lncRNAs and diseases based on various features of diseases and lncRNAs. They introduced a lncRNA–disease relationship prediction method based on DeepWalk. The heterogeneous data was used to build a tripartite network based on three types of nodes. First, they merged heterogeneous data to build an integrated network based on disease–lncRNA, disease–microRNA, and microRNA–lncRNA interactions. Second, the DeepWalk method was used to extract the structure features of the nodes. Third, the similarity scores of disease–disease and lncRNA–lncRNA relationships were calculated based on the network’s topology. Finally, the rule-based inference method discovered new lncRNA and disease associations.

Bonidia et al.^34^ proposed a method to diagnose different lncRNAs cases. They extracted features based on a Fourier transform, using discrete Fourier transform (DFT) with different representations to classify the lncRNAs. Four classification techniques were used to build their system: SVM, random forest (RF), AB, and NB. Wang et al.^35^ discussed how to analyze the relation between lncRNAs and diseases, develop the prediction model, and predict the unknown relations between lncRNAs and diseases. They built a lncRNA–disease association prediction model based on the latent factor model and projection (LFMP). Their model used different data for predicting the unknown relationships between lncRNAs and disease, such as the relationships between miRNA and disease and between miRNA and lncRNA. Their model detected an unknown lncRNA–disease association for lung and colorectal tumors.

**Table 2:** A comparison of some recent studies.

Study	Year	Analysis	Methodology	Dataset
Radivojac et al.^18^	2008	Identifying genes related to disease based on PPI	PPI, SVM	HPRD, Swiss-Prot
		network		
Zhang et al.^23^	2011	Predicting genes related to Parkinson’s disease based on gene expression	PCC, TOPPGene	NCBI GEO
Yang et al.^19^	2014	Predicting disease-genes based on PPI, GO, and gene expression similarity	EPUI	HPRD, OPHID
Peng et al.^30^	2017	Predicting disease-related genes based on genes, diseases, and ontology	SLN-SRW	Clinvar, GO, DO, STRING, OMIM
Hwang^20^	2017	Identifying genes related to disease based on random forests	SRF	OMIM, HPRD, OPHID, GO
Tian et al.^31^	2017	Predicting genes related to disease based on an integrated gene similarity network	RWRB, SNF	swiss-Prot, MimMiner, OMIM, GO, GOA, Pfam
Ding et al.^21^	2018	Predicting lncRNAs genes related to diseases	TPGLDA	LncRNADisease, DisGeNET
Peng et al.^17^	2019	Parkinson’s disease genes prediction based on proteins genes	N2A-SVM	ClinVar
Lei et al.^15^	2019	Predicting disease-related genes based on protein , lncRNAs, and disease	InLPCH	LncRNADisease, HPRD, OMIM
Xuan et al.^22^	2019	Predicting disease related to lncRNA genes	CNNLDA	LncRNADisease, Lnc2Cancer, GeneRIF, starBase, DincRNA
Zhang et al.^33^	2019	Predicting lncRNAs related to disease based on lncRNAs, micoRNA, and diseases	DeepWalk, Rule-based inference	Lnc2Cancer, HMDD, miR2Disease,miRCancer, lncRNADisease
Yang et al.^24^	2020	Predicting disease-related genes based on disease-gene gene-GO, and disease-phenotype	PDGNet	DisGeNet, HPO, OrphaNet, STRING, HPRD, IntAct, PINA,
Bonidia et al.^36^	2020	Diagnosing between different cases lncRNAs	DFT, Entropy, Complex Network	RefSeq, GreeNC Ensembl (v87, v32)
Wang et al.^35^	2021	Identifying lncRNAs related to diseases based on lncRNA, miRNA, and disease	LFMP	MNDRv2.0, MNDRv2.0, Starbase v2.0
Joodaki et al.^32^	2021	Identifying genes related to disease based on similarity network	RWRHN-FF	DisGeNet, OMIM, KEGG, UniProt, GO, Pfam, COXPRESdb
Bi et al.^25^	2021	Predicting PD-related genes and brain regions	CERNNE	PPMI

As mentioned above, the current studies have several limitations, summarized in the following points. First, most studies have developed methods to predict the genes related to diseases, but a few of these methods were designed for PD gene prediction^18–22,32,35^. Second, some of these PD methods identified only protein genes related to PD and ignored lncRNA genes, although lncRNAs are critical for improving our understanding and diagnosing different diseases^17,23–25^. Third, the evaluation measures for identifying disease-related genes are still challenging^15,17,23,30^. Finally, in some studies using deep learning, their models are prone to severe overfitting issues, and the training takes more time and requires large memory^17,22,24,33^.

To overcome these limitations, we designed the prediction system that primarily identifies genes related to PD based on the protein and lncRNA genes to benefit from the biological importance of lncRNAs besides the proteins. The proposed system represents all genes as DNA FASTA sequences to get essential and distinguishing information. We extracted the most significant features of these FASTA sequences based on the PyFeat method with AB as a feature selection technique^29^. The selected features are fed to the GBDT technique to aid in diagnosing different test cases. Finally, for evaluation, seven different performance metrics are applied to validate the proposed system.

## Materials and methods

The main contribution of our system is the identification of PD-related genes: protein and lncRNA, which can aid in the diagnosis and treatment of the disease. The proposed prediction system represents PD genes as DNA FASTA sequences using the University of California Santa Cruz (UCSC) genome browser. We extracted most of the significant features using various feature extraction methods. Based on our experiments, the proposed extracted features based on the PyFeat method with AB contain vital and distinguishing information representing DNA sequences. These features play an essential role in PD-related gene prediction. These selected features are fed to the GBDT technique to diagnose different test cases in our proposed system. Consequently, the proposed system can analyze two separate datasets: proteins and lncRNAs. We used a various performance metrics to validate our system.

**Figure 1:**
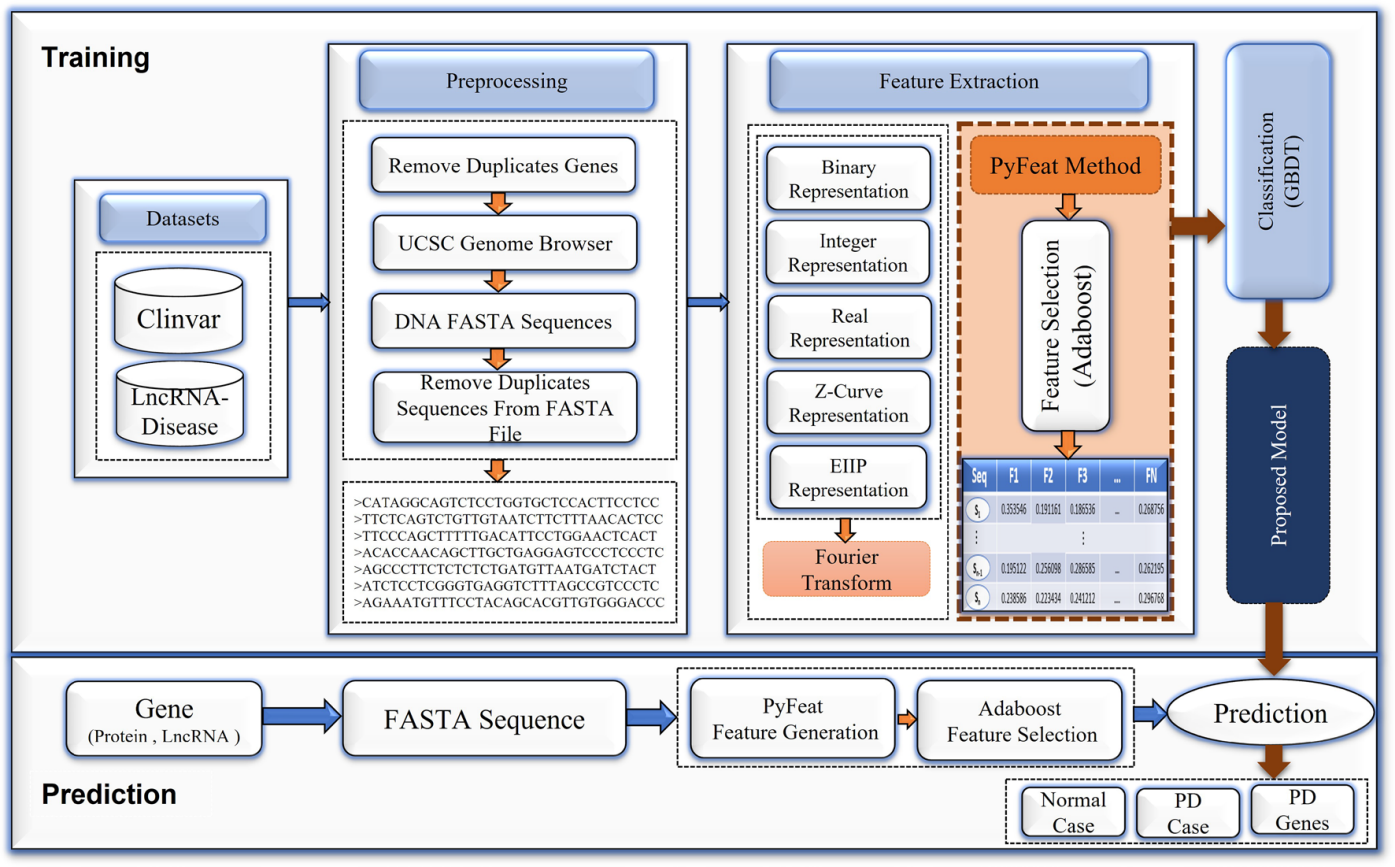
The proposed prediction system for identifying protein and lncRNA genes associated with PD.

Figure 1 shows a novel framework of the proposed prediction system comprising four steps. First, the preprocessing step for removing gene duplication is followed by representing genes as DNA FASTA sequences and removing duplicate sequences from a FASTA file. Second, the most significant features are extracted based on the PyFeat method with the AB technique as a feature selection. Based on our experiments, the proposed features based on the PyFeat with AB achieve promising results compared with state-of-the-art features extraction methods, including Representations Features Fusion (RFF) from five numerical representations with Fourier transform. Third, these proposed features are fed to the GBDT technique to diagnose different test cases. Finally, we evaluate the proposed system results through seven performance measures, which show promising results compared with other systems. The proposed prediction system is detailed in the following subsections.

### Prepossessing

In the preprocessing step, to enhance our proposed system and get accurate results, we prepared and enhanced the original data to feed it to the feature extraction methods. First, the datasets of protein and lncRNA genes were checked, and we noticed repeated genes in these datasets, which we removed. Second, we represented these unique datasets as DNA FASTA sequences and downloaded FASTA files for each protein and lncRNA datasets from the UCSC genome browser^37^. These DNA FASTA sequences contain many significant local and global information about the genes, which aids in extracting the most important feature by using feature extraction techniques. Finally, some sequences are duplicated with the same id in the FASTA files, so the duplicated sequences were identified and removed from these FASTA files using seqkit rmdup^38^.

### Feature extraction

The Feature extraction step aims to reduce the number of features in a dataset by creating new features from the existing ones. These extracted features should be able to summarize most of the information contained in the original data. This step helps in reducing model overfitting, complexity, and computation time. So, we tried to extract the most significant features from the DNA FASTA sequences. Suppose the wrong or unimportant features are used as input to machine learning. In that case, it cannot provide an accurate prediction as the quality of input data is the key to the success of the machine learning model. Therefore, we tried extracting most of the significant features from the DNA FASTA sequences^39,40^. These extracted features help us correctly identify protein and lncRNA genes related to PD. This step is considered a critical step in our proposed prediction system because if the features are not selected properly, the classification might be degraded, undermining the accuracy of the prediction model.

In this section, we described different features extraction methods that achieved promising results compared with state-of-the-art techniques: Pse-in-one2.0^41^, iLearn^42^, and SubFeat^43^. First, we applied the Fourier transform with five numerical mapping representations: binary, integer, real, Z-curve, and electron-ion interaction pseudopotential (EIIP)^34,36^. All extracted features from all representations are fused and referred to as the RFF method. Second, we used PyFeat, which uses 13 biological methods for feature generation, and AB as a feature selection technique. The PyFeat method with AB achieved promising results compared with other methods, including the RFF method. It is important to remember that a biological sequence is defined as $S = (S [0], S [1], \ldots , S [L \ - 1])$ in order for $S\in \big \{ A, C, G, T \big \}$.

#### Fourier transform and numerical mappings

For extracting features, the DFT was applied. It is commonly used in digital image and signal processing fields. DFT can reveal hidden periodicities after translating from the time to frequency domain^36^. It is important to remember that the length of a sequence in the time domain is defined as *L*, the value of the sequence’s element in the time domain is defined as $q[l], \;l=0, 1, \ldots , L-1.$, and *l* is the element’s index in the time domain. For a frequency sequence with length *L* in frequency domain, the frequency element’s value is defined as $Q[f], \;f=0,1,\ldots ,L-1.$, and *f* is the frequency element’s index.

The DFT for a signal with length *L*, is used to calculate *Q*[*f*] at index *f*, as shown in Eq. (1), where $q[l], \;l=1,2,\ldots ,L-1.$ at index *l*.

1 $$\begin{aligned} Q[f]=\sum _{l=0}^{L-1}q[l] e^{-j\frac{2\pi }{L}{fl}},\quad f=0, 2, \ldots ,L-1. \end{aligned}$$

This approach has been extensively investigated in bioinformatics, primarily for studying of recurring elements and periodicities in DNA sequences. To compute DFT for a sequence, we used the fast Fourier transform (FFT), a very effective method for calculating the DFT. Thus, we used five numerical mapping representations: binary, integer, real, Z-curve, and EIIP.

##### Binary representation

This representation can use single or multidimensional vectors. Essentially, this method converts a sequence $S \in \big \{A, C, G, T\big \} ^ L$ into a matrix with size $(4*L)$ as $b \in \big \{0,1\big \} ^{4L}$ such that $b=[b1, b2, b3, b4]^T$ , where T is the transpose operator. The array for each $b_1[l], b_2[l], b_3[l], b_4[l]$ is built using Eq. (2). In this equation, for a binary sequence with length *L* in time domain, the binary element’s value is defined as $b[l], \;l=0,1,\ldots ,L-1.$and *l* is binary element’s index.

2 $$\begin{aligned} b_{i}[l]=\left\{ \begin{array}{ll} 1,S[l]=\alpha [i] \\ 0,S[l]\ne \alpha [i] \end{array} \right. ,\quad \text {where}\quad \alpha =(A, C, G, T),\quad i=(1,2,3,4), \quad l=0,1,\ldots , L-1. \end{aligned}$$

Therefore, in matrix b, each row could be an array that denotes the presence of base A in the first row, base C in the second row, base G in the third row and base T in the fourth row. For example, sequence $S=(T, G, A, C, C, G, A, G, A, G, A)$ is represented using binary form, where $b_1=(0, 0, 1, 0, 0, 0, 1, 0, 1, 0, 1)$ stands for A-bases, $b_2=(0, 0, 0, 1, 1, 0, 0, 0, 0, 0, 0)$ stands for C-bases, $b_3=(0, 1, 0, 0, 0, 1, 0, 1, 0, 1, 0)$ stands for the G-bases, and $b_4=(1, 0, 0, 0, 0, 0, 0, 0, 0, 0, 0)$ stands for T-bases. After that, the DFT is used for these binary sequences with length *L* using Eq. (3), where the frequency element’s value is defined as $B[f], f=0,1,\ldots ,L-1.$ and *f* is the frequency element’s index with $b[l], \;l=1,2,\ldots ,L-1.$ Also, we obtain the power spectrum $P_B [f]$ at index *f* for these binary sequences $B_1[f],B_2[f],B_3[f],B_4[f]$, using Eq. (4).

3 $$\begin{aligned}&B[f]=\sum _{n=0}^{L-1}b[l] e^{-j\frac{2\pi }{L}{fl}},\quad \forall i \in [1,4],\quad f=0, 1, \ldots ,L-1. \end{aligned}$$

4 $$\begin{aligned}&P_B [f]=\sum _{i=1}^4 |B_i [f]|^2,\quad f=0, 1, \ldots ,L-1. \end{aligned}$$

##### Integer representation

This is a one dimensional representation. We convert the four nucleotides of a biological sequence with length *L* (*T*, *C*, *A*, *G*) into integers (0, 1, 2, 3). For instance, sequence $S=(T, G, A, C, C, G, A, G, A, G, A)$ with length *L* is represented as $g=(0, 3, 2, 1, 1, 3, 2, 3, 2, 3, 2)$, which is defined using Eq. (5), where the integer element’s value in time domain is defined as $g[l], l=0,1,\ldots ,L-1.$, at index *l*. Then, the DFT and power spectrum $P_G[f]$ of the integer sequence are defined using Eq. (6), where the frequency element’s value is defined as $G[f], f=0,1,\ldots ,L-1.$ and *f* is the frequency element’s index with $g[l], l=1,2,\ldots ,L-1.$

5 $$\begin{aligned}&g[l]=\left\{ \begin{array}{ll} 0,\;\;S[l]=T\\ 1,\;\;S[l]=C \\ 2,\;\;S[l]=A \\ 3,\;\;S[l]=G \\ \end{array} \right. ,\quad l=0, 1, \ldots ,L-1. \end{aligned}$$

6 $$\begin{aligned}&G[f]=\sum _{l=0}^{L-1}g[l] e^{-j\frac{2\pi }{L}{fl}}, \quad P_G[f]= |G [f]|^2,\quad f=0, 1, \ldots ,L-1. \end{aligned}$$

##### Real representation

This representation uses the complement property of the complex mapping for real number representation^44^. The real representation is $-\,1.5$ for A, $-\,0.5$ for G, 0.5 for C, and 1.5 for T, as represented using Eq. (7), where the real element’s value is defined as $r[l], \;l=0,1,\ldots ,L-1.$ and *l* is the real element’s index in time domain. For example, sequence $S=(T, G, A, C, C, G, A, G, A, G, A)$ is represented as $r=(1.5, -\,0.5, -\,1.5, 0.5, 0.5, -\,0.5, -\,1.5, -\,0.5, -\,1.5, -\,0.5, -\,1.5)$. The DFT and power spectrum $P_R[f]$ of the real sequence are defined using Eq. (8), where the frequency element’s value is defined as $R[f], \;f=0,1,\ldots ,L-1.$ and *f* is the frequency element’s index with $r[l], \;l=1,2,\ldots ,L-1.$

7 $$\begin{aligned}&r[l]=\left\{ \begin{array}{ll} -\,1.5,\;\;S[l]=A \\ -\,0.5,\;\;S[l]=G \\ 0.5,\;\;S[l]=C \\ 1.5,\;\;S[l]=T \end{array} \right. ,\quad l=0, 1, \ldots ,L-1. \end{aligned}$$

8 $$\begin{aligned}&R[f]=\sum _{l=0}^{L-1}r[l] e^{-j\frac{2\pi }{L}{fl}},\quad P_R[f]=|R {[}f]|^2,\quad f=0, 1, \ldots ,L-1. \end{aligned}$$

##### Z-curve representation

This three-dimensional curve, is used to describe DNA sequences with biological meaning. We can check sequence *S*[*l*] with length *L*, considering the l-th element of the sequence $(l=1, 2, \ldots , L).$ After that, we use the aggregate appearance numbers for each base $A_l, C_l, G_l$, and $T_l$, representing the frequency of a base’s presence from *S*[1] to *S*[*L*]. Using this method, we reduce the number of indications for sequences from four to three for all four elements symmetrically way^45^.

9 $$\begin{aligned} A_l + C_l + G_l +T_l=l \end{aligned}$$

This Z-curve is made from a set of nodes $P_1, P_2, \ldots , P_L$, which the coordination *x*[*l*], *y*[*l*],  and *z*[*l*], where $(l=1, 2, \ldots , L)$ defined exclusively based on the Z-transform, as shown in Eq. (10).

10 $$\begin{aligned} P[l]=\left\{ \begin{array}{ll} x[l]=(A_l+G_l)-(C_l+T_l)\\ y[l]=(A_l+C_l)-(G_l+T_l)\\ z[l]=(A_l+T_l)-(C_l+G_l) \end{array} \right. ,\quad x[l], y[l], z[l] \in [ -l,l],\quad l = 0, 1,\ldots , L. \end{aligned}$$

The distributions *x*[*l*], *y*[*l*],  and *z*[*l*] fully describe a sequence. Consequently, three biologically significant distributions will be available: (1) *x*[*l*]= purine/pyrimidine, (2) *y*[*l*]= amino/keto, (3) *z*[*l*]= weak hydrogen bonds/strong hydrogen bonds. For example, sequence $S=(T, G, A, C, C, G, A, G, A, G, A)$, will be represented with three distributions: $x=(-\,1, 0, 1, 0, -\,1, 0, 1, 2, 3, 4, 5),$ $y=(-\,1, -\,2, -\,1, 0, , 0, 1, 0, 1, 0, 1),$ $z=(1, 0, 1, 0, -\,1, -\,2, -\,1, -\,2, -\,1, -\,2, -\,1).$ After that, the DFT and the power spectrum $P_P[f]$ are defined using Eqs. (11) and (12). In these equations, for Z-curve sequence *P*[*l*] with length *L*, for each *x*[*l*], *y*[*l*], *z*[*l*], the elements’ values for *x* are defined as $x[l], \;l=0,1,\ldots ,L-1.$ and *l* is the element’s index in time domain, similarity for *y*[*l*], *z*[*l*]. For each *X*[*f*], *Y*[*f*], *Z*[*f*], the frequency elements’ values for *X* are defined as $X[f], \;f=0,1,\ldots ,L-1.$ and *f* is the frequency element’s index, similarity for *Y*[*f*], *Z*[*f*].

11 $$\begin{aligned}&X[f]=\sum _{l=0}^{L-1}x[l] e^{-j\frac{2\pi }{L}{fl}},\quad Y[f]=\sum _{l=0}^{L-1}y[l] e^{-j\frac{2\pi }{L}{fl}},\quad Z[f]=\sum _{l=0}^{L-1}z[l] e^{-j\frac{2\pi }{L}{fl}} \end{aligned}$$

12 $$\begin{aligned}&P_P[f]=|X [f]|^2 + |Y [f]|^2 + |Z [f]|^2,\quad f=0, 1, \ldots ,L-1. \end{aligned}$$

##### EIIP representation

EIIP values of nucleotides for representing DNA sequences and for locating exons were proposed in^46^. According to this study, the EIIP representation is 0.0806 for G, 0.1260 for A, 0.1335 for T, and 0.1340 for C, as shown in Eq. (13), where the EIIP element’s value is defined as $d[l], \;l=0,1,\ldots ,L-1.$ and *l* is the EIIP element’s index in time domain. For example, $S=(T, G, A, C, C, G, A, G, A, G, A)$ is represented as $d=(0.1335, 0.0806, 0.1260, 0.1340, 0.1340, 0.0806, 0.1260, 0.0806, 0.1260, 0.0806, 0.1260).$ The DFT and power spectrum $P_D[f]$ of this representation are defined using Eq. (14), where the frequency element’s value is defined as $D[f], \;f=0,1,\ldots ,L-1.$ and *f* is the frequency element’s index with $d[l], \;l=1,2,\ldots ,L-1$.

13 $$\begin{aligned}&d[l]=\left\{ \begin{array}{ll} 0.0806,\;\;S[l]=G \\ 0.1260,\;\;S[l]=A \\ 0.1335,\;\;S[l]=T\\ 0.1340,\;\;S[l]=C \end{array} \right. ,\quad l=0, 1, \ldots ,L-1. \end{aligned}$$

14 $$\begin{aligned}&D[f]=\sum _{l=0}^{L-1}d[l] d^{-j\frac{2\pi }{L}{fl}},\quad P_D[f]=|D [f]|^2,\quad f=0, 1, \ldots ,L-1. \end{aligned}$$

##### Features

We used the feature extraction for each representation depending on the peak to average power ratio (PAPR), signal to noise ratio (SNR), minimum, maximum, median, population standard deviation, sample standard deviation, percentile (15/25/50/75), variance, coefficient of variation, amplitude, semi-interquartile range, interquartile range, skewness, and kurtosis^34,36^.

#### PyFeat

Extracting crucial features is essential in representing biological DNA sequences and identifying genes related to disease. The PyFeat is used to create different numeric feature representations for biological sequences. Additionally, it can be used to describe the fusion of essential features from broad neighboring residues. It focuses on extracting features that collect information about the relationships of neighboring residues so that more local and global features can be provided. This method can also choose the best and most essential features from a set of features created primarily by the gap.

We have selected a group of features from different methods for biological DNA sequences: Z-curve, gcContent, cumulative skew, Chou’s pseudo composition, monoMonoKGap, monoDiKGap, monoTriKGap, diMonoKGap, diDiKGap, diTriKGap, triMonoKGap, and triDiKGap^29^. After the feature generation, the AB technique was used to select features with the most discriminatory information possible to reduce the dimensionality, complexity, and computational time. Thus, the number of features extracted can be reduced significantly. We used the PyFeat to represent the combination of essential features from large neighboring residues.

**Features generation** This intends on catching the frequency distributions of different permutations of the base nucleotide acids in biological DNA sequences. It is used to describe the sequences in the model training phase based on the kGap. For DNA sequences, when the value of kGap is small, the number of generated features is also small, and the occurrence frequency of the generated features keeps local or short-range sequence-order information. However, if the value of kGap is moderately large, the generated features maintain global or long-range sequence-order information. According to the previous analysis, we consider the features where kGap values are equal to five to extract features that include local and global information. Table 3 shows the most significant features that are extracted from these different methods.

**Z-curve** It is often used in genomic sequence analysis. It has three components on three axes. They are defined using Eq. (13), where three features are generated based on the Z-curve method.

**GCcontent** This measure shows the proportion of G and C elements out of four elements (A, C, G, and T) in a sequence. It is defined using Eq. (15).

15 $$\begin{aligned} GC=\frac{\sum G +\sum C}{\sum A +\sum C +\sum G +\sum T}\times 100\% \end{aligned}$$

**ATGC ratio** This represents the summation ratio of the A and T elements to the summation of the G and C elements in a DNA sequence. It is defined using Eq. (16).

16 $$\begin{aligned} ATGC Ratio=\frac{\sum A +\sum T}{\sum G + \sum C} \end{aligned}$$

**Cumulative skew** This considers two measures as the GC skew and AT skew. The GC skew is determined as the normalized excess of G and C in a sequence. Similarly, AT skew is determined as the normalized excess of A and T in a sequence, as defined using Eq. (17).

17 $$\begin{aligned} GC skew=\frac{\sum G -\sum C}{\sum C - \sum G};\;\;AT skew=\frac{\sum A -\sum T}{\sum T - \sum A} \end{aligned}$$

**Pseudo composition** This measure determines the frequencies of subsequences, where *n* is the subsequences length. The number of generated features based on the Pseudo Composition method from a sequence is defined as *num*(*PC*), as shown in Eq. (18). In this equation and the following equations 4 is a sequence elements (A, C, G, T), and *K* is the length of the longest subsequence. $K=3$, then only 84 features exist. These features are determined by the frequencies of subsequences: $A, C, G, T, AA, \dots , TT, AAA, \dots , and\; TTT$ in the whole DNA sequence.

18 $$\begin{aligned} num(PC)=\sum _{o=1}^{K} (4^k),\quad o=1, 2, \ldots ,K. \end{aligned}$$

**monoMonoKGap** The generated features are determined based on the frequencies of subsequences with single nucleotides at the beginning and end and number of Gaps (*kGap*) between them. The number of generated features based on the monoMonoKGap method for the DNA sequence is defined as *num*(*MM*), as shown in Eq. (19),where *n* is the length of the longest *kGap*. $n=5$, then only 80 features exist. These features are determined by the frequencies of subsequences: $A-A, \ldots , T-T, A- -A, \ldots , T- -T, A- - -A,\ldots , T- - -T, A- - - -A, \ldots , T- - - -T, A- - - - -A, \ldots , and\; T- - - - -T$ in the whole DNA sequence.

19 $$\begin{aligned} num (MM)=4\times 4\times n \end{aligned}$$

**monoDiKGap** The generated features are extracted based on the frequencies of subsequences with single nucleotide at the beginning and two nucleotides at the ends and *kGap* between them. The number of generated features based on the monoDiKGap method for the DNA sequence is defined as *num*(*MD*), as shown in Eq. (20), where *n* is the length of the longest *kGap*. $n=5$, then 320 features exist. These features are determined by the frequencies of subsequences: $A-AA, \ldots , T-TT, A- -AA, \ldots , T- -TT, A- - -AA, \ldots , T- - -TT, A- - - -AA, \ldots , T- - - -TT, A- - - - -AA, \ldots , and\; T- - - - -TT$ in the whole DNA sequence.

20 $$\begin{aligned} num (MD)=(4)\times (4 \times 4) \times n \end{aligned}$$

**monoTriKGap** The generated features are extracted based on the frequencies of subsequences with single nucleotide at the beginning and three nucleotides at the ends and *kGap* between them. The number of generated features based on the monoTriKGap method for the DNA sequence is defined as *num*(*MT*), as shown in Eq. (21), where *n* is the length of the longest *kGap*. $n=5$, then 1280 features exist. These features are determined by the frequencies of subsequences: $A-AAA, \ldots , T-TTT, A- -AAA, \ldots , T- -TTT, A- - -AAA, \ldots , T- - -TTT, A- - - -AAA, \ldots , T- - - -TTT, A- - - - -AAA, \ldots , and \;T- - - - -TTT$ in the whole DNA sequence.

21 $$\begin{aligned} num(MT)=(4)\times (4\times 4\times 4)\times n \end{aligned}$$

**diMonoKGap** The generated features are extracted based on the frequencies of subsequences with two nucleotides at the beginning and single nucleotide at the ends and *kGap* between them where $kGap=n$. The number of generated features based on the diMonoKGap method for DNA sequence is defined as *num*(*DM*), as shown in Eq. (22), where *n* is the length of the longest *kGap*. $n=5$, then 320 features exist. These features are determined by the frequencies of subsequences: $AA-A, \ldots , TT-T, AA- -A, \ldots , TT- -T, AA- - -A, \ldots , TT- - -T, AA- - - -A, \ldots , TT- - - -T, AA- - - - -A, \ldots , and \;TT- - - - -T$ in the whole DNA sequence.

22 $$\begin{aligned} num(DM)=(4\times 4)\times (4)\times n \end{aligned}$$

**diDiKGap** The generated features are extracted based on the frequencies of subsequences with two nucleotides at the beginning and two nucleotides at the ends and *kGap* between them. The number of generated features based on the diDiKGap for the DNA sequence is defined as *num*(*DD*), as shown in Eq. (23), where *n* is the length of the longest *kGap*. $n=5$, then 1280 features exist. These features are determined by the frequencies of subsequences: $AA-AA, \ldots , TT-TT, AA- -AA, \ldots , TT- -TT, AA- - -AA, \ldots , TT- - -TT, AA- - - -AA, \ldots , TT- - - -TT, AA- - - - -AA, \ldots , and\; TT- - - - -TT$ in the whole DNA sequence.

23 $$\begin{aligned} num(DD)=(4\times 4)\times (4\times 4)\times n \end{aligned}$$

**diTriKGap** The generated features are extracted based on the frequencies of subsequences with two nucleotides at the beginning and three nucleotides at the ends and *kGap* between them where $kGap=n$. The number of generated features based on the diTriKGap method for the DNA sequence is defined as *num*(*DT*), as shown in Eq. (24), where *n* is the length of the longest *kGap*. $n=5$, then 5120 features are existed, these features are determined by the frequencies of subsequences: $AA -AAA, \ldots , TT-TTT, AA- -AAA, \ldots , TT- -TTT, AA- - -AAA, \ldots , TT- - -TTT, AA- - - -AAA, \ldots , TT- - - -TTT, AA- - - - -AAA, \ldots , and \;TT- - - - -TTT$ in the whole DNA sequence.

24 $$\begin{aligned} num(DT)=(4\times 4)\times (4\times 4\times 4)\times n \end{aligned}$$

**triMonoKGap** The generated features are extracted based on the frequencies of subsequences with three nucleotides at the beginning and single nucleotide at the ends and *kGap* between them where $kGap=n$. The number of generated features based on the triMonoKGap method for the DNA sequence is defined as *num*(*TM*), as shown in Eq. (25), where *n* is the length of the longest *kGap*. $n=5$, then 1280 features are existed, these features are determined by the frequencies of subsequences: $AAA-A, \ldots , TTT-T, AAA- -A, \ldots , TTT- -T, AAA- - -A, \ldots , TTT- - -T, AAA- - - -A, \ldots , TTT- - - -T, AAA- - - - -A, \ldots , and \;TTT- - - - -T$ in the whole DNA sequence.

25 $$\begin{aligned} num(TM)=(4\times 4\times 4)\times (4)\times n \end{aligned}$$

**triDiKGap** The generated features are extracted based on the frequencies of subsequences with three nucleotides at the beginning and two nucleotides at the ends and *kGap* between them where $kGap=n$. The number of generated features based on the triDiKGap method for the DNA sequence is defined as *num*(*TD*), as shown in Eq. (26), where *n* is the length of the longest *kGap*. $n=5$, then 5120 features exist. These features are determined by the frequencies of subsequences: $AAA-AA, \ldots , TTT-TT, AAA- -AA, \ldots , TTT- -TT, AAA- - -AA, \ldots , TTT- - -TT, AAA- - - -AA, \ldots , TTT- - - -TT, AAA- - - - -AA, \ldots , and \;TTT- - - - -TT$ in the whole DNA sequence. Table 3 shows the overall methods utilized by PyFeat and the number of features for each method.

26 $$\begin{aligned} num(TD)=(4\times 4\times 4)\times (4\times 4)\times n \end{aligned}$$

**Feature selection** Different methods are used based on the PyFeat feature generation: Z-curve, gcContent, ACGT ratio, Cumulative Skew, Chou’s Pseudo composition, monoMonoKGap, monoDiKGap, monoTriKGap, diMonoKGap, diDiKGap, diTriKGap, triMonoKGap, and triDiKGap. Thus, we obtained many features for each biological sequence, as shown in Table 3.

**Table 3:** PyFeat feature generation and their numbers.

Method	Number of features
Z-curve	3
GCcontent	1
ATGC ratio	1
Cumulative Skew	2
Pseudo composition	84
monoMonoKGap	80
monoDiKGap	320
monoTriKGap	1280
diMonoKGap	320
diDiKGap	1280
diTriKGap	5120
triMonoKGap	1280
triDiKGap	5120
# of features	14,891

To reduce the complexity and computational time for the classifier, the AB technique is used to reduce the feature vector dimension obtained using the PyFeat method and concurrently keep informative features. AB technique achieves an average impurity-curtailment, according to dividing each feature on all the trees trained based on various weight distributions. Thus, the features with the maximum score in the trained model are selected using the real-valued School of Aerospace, Mechanical and Manufacturing Engineering (SAMME.R) algorithm^47^. We use the SAMME.R algorithm as feature selection to select n features with the maximum score in the trained model according to these composite features. After applying the SAMME.R, we obtain 213 features as average for each biological sequence instead of 14,891 features generated by PyFeat^29^, as shown in Table 3. We represent the algorithm for the proposed preprocessing and feature extraction technique using PyFeat with the AB technique as feature selection, as shown in Algorithm 1.
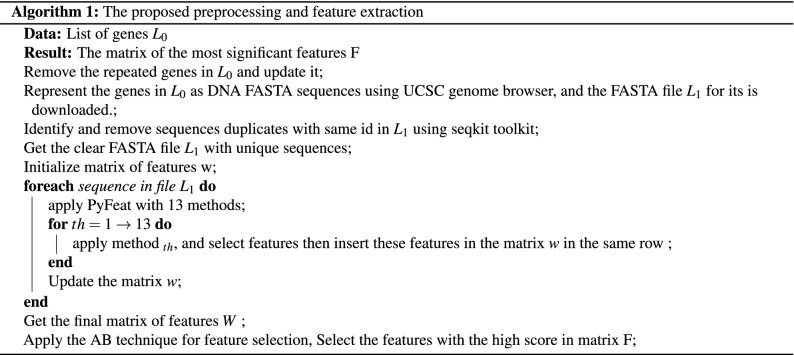


### Classification

The features of the DNA sequence are fed to the GBDT technique. This technique is used to diagnose different test cases and predict the protein and lncRNA genes related to PD. Our experiments show that the GBDT is better than state-of-the-art machine-learning techniques, which are used for classification and regression problems. The final result achieved according to the summation of all trees’ consequences was established from several decision trees. Via numerous iteration rounds, weak classifiers were generated in each GBDT iteration, and each classifier was trained based on the gradient of classifiers in the previous round. The final classifier is identified based on the summation of weights for the weak classifiers, which are resulted in each round of training^48–50^. The model training is shown in the subsequent steps:

1. The initialized predicted value for all samples (x), is defined as $h_0(x)$ model as shown in Eq. (27).
  27 $$\begin{aligned} h_0(x)=0.5*log \left( \frac{\sum _{i=1}^{N} y_i}{\sum _{i=1}^{N} 1- y_i}\right) \end{aligned}$$
  where N is the number of samples in training set, $y_i$ is the real label of each sample.
2. The loss between a predicted value $h_{m}(x_i)$ in the $m_{th}$ round and a real value $y_i$ is defined as the loss function $F(y_i, h_{m}(x_i))$ for each sample $x_i$, as shown in Eq. (28).
  28 $$\begin{aligned} F(y_i, h_{m-1}(x_i))= log(1+exp(-y_i h_{m-1}(x_i))) \end{aligned}$$
3. For each round where $m={1,2, \ldots , M}$
  - For $i_{th}$ sample in $m_{th}$ round, the negative gradient “pseudo residuals” $r_{m,i}$ of the loss function as defined using Eq. (29).
    29 $$\begin{aligned} r_{m,i}=\frac{y_i}{1+ (exp \;\;(y_i)\;\; h(x_i))},\quad i= 1, 2, \ldots , N. \end{aligned}$$
  - Fit a regression tree $m_{th}$ to the $r_{m,i}$ values to create the terminal regions “tree leaf nodes” $R_{m,j}$ with one or multiple $r_{m,i}$, where $j=1,2,\ldots ,J$, which J is the number of leaf nodes in the tree.
  - The optimal outcome value of fitting the leaf node $(v_{m,j})$ for samples in each terminal region “leaf node”, which minimizes the loss function, given by is calculated using Eq. (30).
    30 $$\begin{aligned} v_{m,j}= arg \mathop {min}_v \sum _{x \in R_{m,j}}{}log(1+exp(-y_i h(x_i)+v) \end{aligned}$$
  - Update $m_{th}$ weak model using Eq. (31).
    31 $$\begin{aligned} h_m=h_{m-1}(x)+ lr *\sum _{j=1}^{J} v_{m,j}I(x\in R_{m,j}) \end{aligned}$$
    where lr is the learning rate with $0<lr\le 1$, and $I(x\in R_{m,j})$ means that if x falls on the leaf node according to $R_{m,j}$, so that this corresponding term is equal 1.
  - See whether M is lower than m. If M is more than m, then go to step (4) to finish the training. Otherwise, go to the step (1) for the next iteration.
4. The end of training with model *H*.
  32 $$\begin{aligned} H(x)=h_{0}(x)+ lr *\sum _{m=1}^{M}\sum _{j=1}^{J} v_{m,j}I(x\in R_{m,j}) \end{aligned}$$

We represent the algorithm for the proposed classification based on the GBDT technique as shown in Algorithm 2.
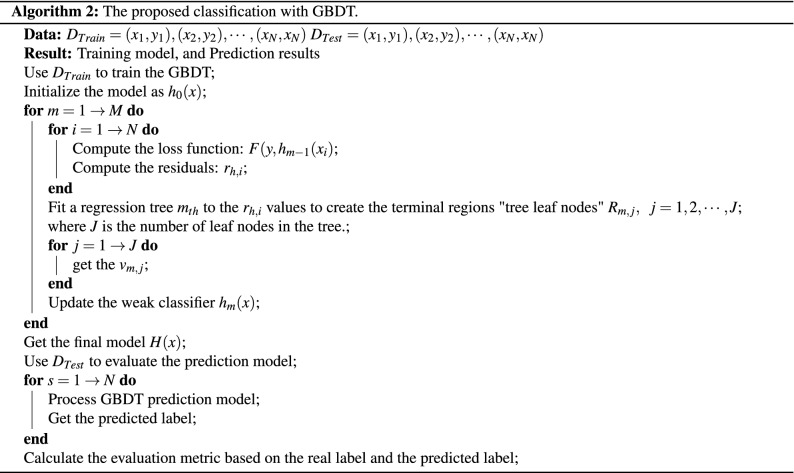


In the end of this section, the important variables, parameters, and symbols of the used formulas are listed in Table 4.

**Table 4:** Definition of important variables, parameters, and symbols of formulas.

S	Biological sequence of element values: A, C, G, T	L	Length of a sequence
*l*	Index of an element in a sequence for time domain	*q*[*l*]	Value of an element at index l in time domain
*f*	Index of an element in a sequence for frequency domain	*Q*[*f*]	Value of an element at index f in frequency domain
*b*	The binary matrix with size (4*L) for [b1, b2, b3, b4]	*b*1	Binary sequence for presenting A element
*b*2	Binary sequence for presenting C element	*b*3	Binary sequence for presenting G element
*b*4	Binary sequence for presenting T element	*b*[*l*]	Binary value of an element at index l in time domain
*B*[*f*]	Frequency value of an element at index f for binary sequence	$P_B[f]$	Power spectrum for B[f]
*i*	Integer representation sequence	*i*[*l*]	Integer value of an element at index l in time domain
*I*[*f*]	Frequency value of an element at index f for integer sequence	$P_I[f]$	Power spectrum for I[f]
*r*	Real representation sequence	*r*[*l*]	Real value of an element at index l in time domain
*R*[*f*]	Frequency element’s value at index f for real sequence	$P_R[f]$	Power spectrum for r[f]
*x*[*l*]	Element’s value for x-coordination of Z-curve at index l in time domain	*y*[*l*]	Element’s value for y-coordination of Z-curve at index l in time domain
*z*[*l*]	Element’s value for z-coordination of Z-curve at index l in time domain	*p*[*l*]	The Z-curve element’s value of x[l], y[l], and z[l] at index l in time domain
*X*[*f*]	Frequency value of an element at index f for x-coordination of Z-curve	*Y*[*f*]	Frequency value of an element at index f for y-coordination of Z-curve
*Z*[*f*]	Frequency value of an element at index for z-coordination of Z-curve	$P_P[f]$	Power spectrum for x[l], y[l], and z[l]
*d*	EIIP representation sequence	*i*[*l*]	EIIP value of an element at index l in time domain
*D*[*f*]	Frequency value of an element at index f for EIIP sequence	$P_D[f]$	Power spectrum for D[f]
*K*	Length of the longest subsequence for pseudo composition method	*num*(*pc*)	Number of features extracted based on pseudo composition method
*KGap*	The number of Gap between nucleotides	*n*	The length of the longest KGap
*num*(*MM*)	Number of features extracted based on the monoMonoKGap method	*num*(*MD*)	Number of features extracted based on the monoDiKGap method
*num*(*MT*)	Number of features extracted based on the monoTriKGap method	*num*(*DM*)	Number of features extracted based on the diMonoKGap method
*num*(*DD*)	Number of features extracted based on the diDiKGap method	*num*(*DT*)	Number of features extracted based on the diTriKGap method
*num*(*TM*)	Number of features extracted based on the triMonoKGap method	*num*(*TD*)	Number of features extracted based on the triDiKGap method
*N*	Number of samples in dataset	$i_{th}$	The id of sample in dataset
$x_i$	The sample with id $i_th$	$y_i$	The real label for sample $x_i$
$h_0(x)$	The initialized predicted value for all samples x, namely, initialized model	$m_{th}$	The id of the round or tree
$F(y_i, h_{m}(x_i))$	Loss function between a predicted value $h_{m}(x_i)$ in the $m_{th}$ round and a real value $y_i$	*M*	Number of rounds in training
$r_{m,i}$	Pseudo residuals or negative gradient of the loss function for $i_{th}$ sample in $m_{th}$ round	*j*	Number of terminal nodes at $m_th$ tree
$R_{m,j}$	Tree leaf node or terminal region with one or multiple $r_{m,i}$	*H*	Final model at the end of training
$(v_{m,j})$	Optimal output value of fitting the leaf node for samples in each leaf node	*lr*	Learning rate with $0<lr\le 1$

## Experimental results

This section represents the datasets description, hardware and software specifications, evaluation metrics, results, and discussion. In the results subsection, first, we extracted the most significant features using the PyFeat method with the AB feature selection technique based on protein and lncRNA datasets. These features achieved promising results compared with features from state-of-the-art feature extraction methods: five numerical representations with Fourier transform, FRR, Pse-in-One2.0, iLearn, and SubFeat. Second, the GBDT classifier is used to build the overall proposed system with the PyFeat method and AB based on protein and lncRNA datasets and compared with state-of-the-art classification algorithms to validate the performance of the GBDT.

Third, the proposed prediction model based on the PyFeat method with AB and GBDT classifier is compared with state-of-the-art systems. After that, we represent some tables and figures supporting a target idea by employing seven performance metrics. Finally, we present an objective comparison of the proposed system with some literature studies in the discussion subsection. Also, we provide the strengths and weaknesses of the proposed system. Furthermore, a literature study can be used to verify the top-ranked predicted protein and lncRNA genes.

### Datasets description

This subsection describes the two utilized datasets: proteins and lncRNAs.

- Protein dataset^17,51^: From the ClinVar, we downloaded protein genes associated with PD. After removing repeated genes, we got 182 genes associated with PD as a positive case. Also, the negative genes not associated with PD are divided into four batches with the size of 185 genes, as shown in Table 5.
- LncRNA dataset^52^: We downloaded lncRNAs genes associated with PD from the LncRNADisease v2.0. We got 137 genes associated with PD as a positive case. Also, the negative genes not associated with PD are divided into eight batches with the size of 141 genes, as shown in Table 5.

**Table 5:** Datasets description.

Datasets	Site	Positive	Negative
Protein	ClinVar	182	185
LncRNA	LncRNADisease v2.0	137	141

### Hardware and software specifications

This subsection describes the specifications of the used software/hardware in our research. We developed this work using Python 3.7.6 and PyCharm 2019.3.3 with pandas, itertools, numpy, sklearn, and matplotlib libraries. We ran our system on a machine of core i7/4.5. It has 16 GB RAM and an NVIDIA GeForce GTX with 4 GB VRAM.

### Evaluation metrics

We used seven metrics for measuring the performance of our proposed system, including accuracy (ACC), area under the curve (AUC), area under precision-recall curve (AUPR), F1-Score, Matthews correlation coefficient (MCC), sensitivity (SEN), and specificity (SPC)^53,54^, which are defined using Eqs. (33)–(41).

33 $$\begin{aligned}&ACC=\frac{TN + TP}{TN+FP+TP+FN} \end{aligned}$$

34 $$\begin{aligned}&Precision=\frac{TP}{FP + TP} \end{aligned}$$

35 $$\begin{aligned}&Recall= SEN= TPR=\frac{TP}{FN +TP} \end{aligned}$$

36 $$\begin{aligned}&F1-score=\frac{TP}{TP+0.5(FN + FP)} \end{aligned}$$

37 $$\begin{aligned}&MCC=\frac{TP.TN - FP.FN }{\sqrt{(FP + TP).(FN + TP).(FP + TN).(FN+TN)}} \end{aligned}$$

38 $$\begin{aligned}&SPC=TNR=\frac{TN}{FP +TN} \end{aligned}$$

39 $$\begin{aligned}&FPR=1-TNR=\frac{FP}{FP +TN} \end{aligned}$$

40 $$\begin{aligned}&AUC=\int _0^1{TPR\;\; d(FPR)} \end{aligned}$$

41 $$\begin{aligned}&\text {AUPR} = \sum _n (Recall_n - Recall_{n-1}) Precision_n \end{aligned}$$

It is essential to clarify that true positive (TP) is the rate of the genes that are correctly predicted as PD-genes. True negative (TN) is the rate of the genes that are correctly predicted as not PD-genes. False positive (FP) is the rate of the genes that are incorrectly predicted as PD genes. Moreover, false negative (FN) is the rate of the genes that are incorrectly predicted as not PD-genes. ACC is the rate of the correct result over the total results based on TP and TN. It determines the proposed system’s accuracy.

The precision is the rate of the correct predicted results over the amount of correct and incorrect prediction results, where the term “results” refers to the positive genes. The SEN or recall or TPR is the rate of the correct predicted results over the all correct predicted results, where the term “results” refers to the negative genes. AUC summarizes the receiver operating characteristic (ROC) curve based on the true positive rate (TPR) and false positive rate (FPR) at different classification thresholds^55,56^. A higher value of AUC gives the best performance when distinguishing between positive and negative PD genes.

AUPR summarizes the precision-recall (PR) curve as the weighted mean of precisions achieved at each threshold and the increase in recall from its previous one used as the weighted measure^57^. The MCC is considered a contingency matrix method to calculate the Pearson product-moment correlation coefficient between actual and predicted values. SPC is the rate of the correct predicted results over the all correct predicted results, where the term “results” refers to the negative genes.

### Results

In this subsection, we present all the experimental results achieved in this study and relevant analysis. The experimental results consisted of three parts: features extraction comparison, classification algorithms comparison, and comparison with other prediction systems. For the protein dataset, the result is the average performance of four negative batches with the positive data. Similarly, for the lncRNA dataset, the result is the average performance of eight negative batches with the positive data.

#### Features extraction comparison

For extracting the important features from DNA FASTA sequences, we used the PyFeat method with AB to build our prediction system. To validate the proposed features, its compared with features from eight state-of-the-art features extraction techniques: five representations with Fourier transform, RFF, Pse-in-one2.0^41^, iLearn^42^, and SubFeat^43^. We preformed the experiments based on protein and lncRNA datasets using the GBDT classifier with 10-Fold cross-validation technique.

The proposed features based on the Pyfeat method with AB achieved promising results compared with other methods. After the proposed features, the features from the RFF method show better results than the remaining methods: five representations with Fourier transform, Pse-in-one2.0, iLearn, and SubFeat. We evaluated the results using seven performance metrics: ACC, AUC, AUPR, F1-score, MCC, SEN, and SPC.

**Protein dataset** Table 6 shows the performance comparison of the proposed features based on PyFeat with AB and features from state-of-the-art features extraction techniques: five representations with Fourier transform, RFF, Pse-in-one2.0, iLearn, and SubFeat. For 10-fold cross-validation, the proposed features achieved the following: ACC equals 79.4%, AUC equals 84.9%, AUPR equals 86.0%, F1-score equals 78.7%, MCC equals 0.590, SEN equals 76.8%, and SPC equals 82.1%. The proposed features based on PyFeat and AB achieve promising results compared with other techniques with the seven performance measures based on the protein dataset.

**Figure 2:**
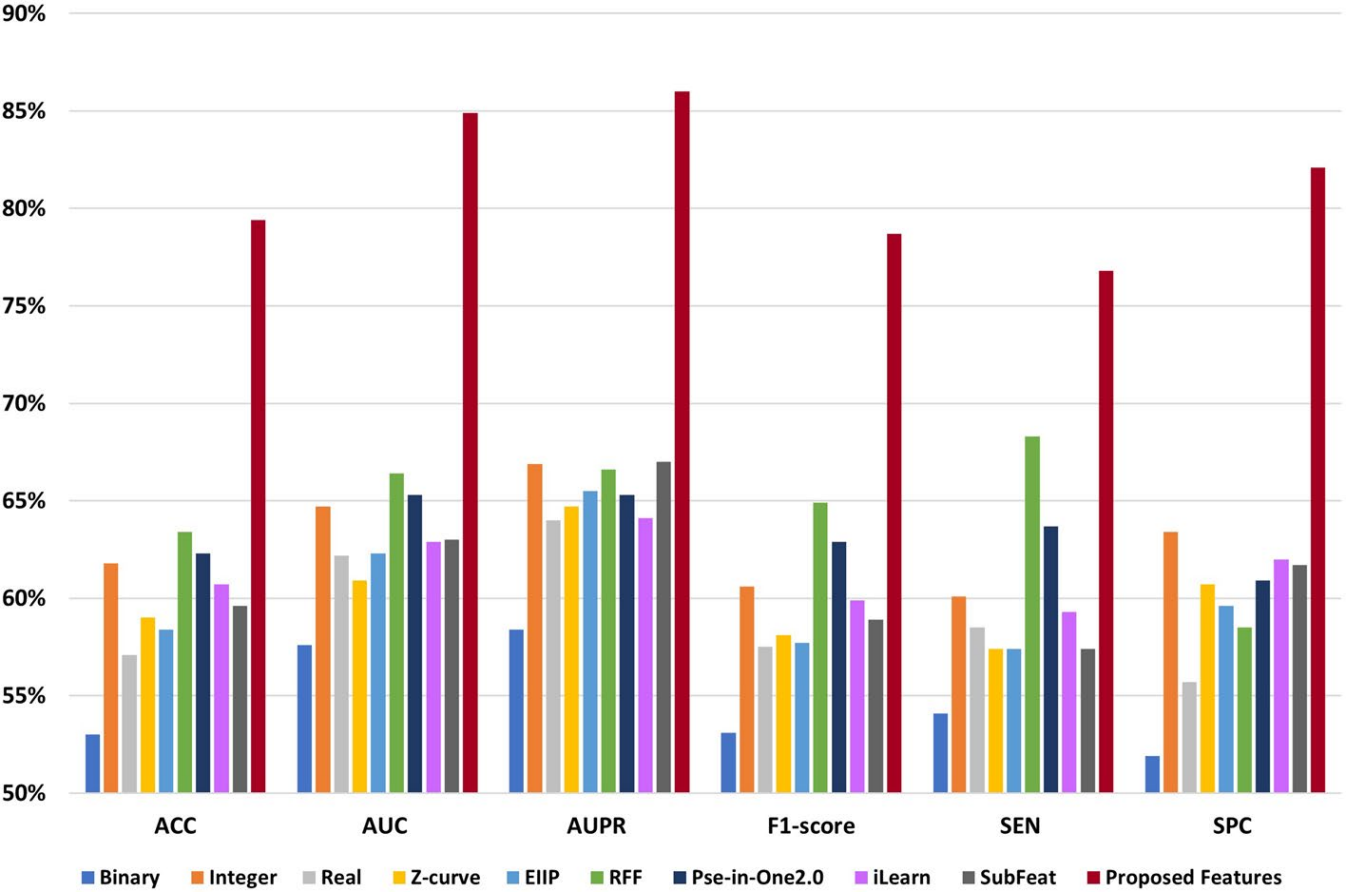
The performance evaluation of the features based on PyFeat with AB compared with other techniques: five numerical representations, RFF, Pse-in-One2.0, iLearn, and SubFeat based on the protein dataset.

After the proposed features, the features based on the RFF method achieve better results than the remaining methods: five representations with Fourier transform, Pse-in-one2.0, iLearn, and SubFeat. Meanwhile, the features based on the binary method give the worst results compared with other methods. Figure 2 represents the comparison chart among performance measures of the features based on PyFeat with AB and other methods on the protein dataset.

**Table 6:** The performance evaluation of the proposed features based on PyFeat with AB compared with other techniques: five numerical representations, RFF, Pse-in-One2.0, iLearn, and SubFeat with 10-fold cross-validation based on the protein dataset. Significant values are in bold.

Metric	ACC (%)	AUC (%)	AUPR (%)	F1-score (%)	MCC	SEN (%)	SPC (%)
Binary	53.0	57.6	58.4	53.1	0.061	54.1	51.9
Integer	61.8	64.7	66.9	60.6	0.241	60.1	63.4
Real	57.1	62.2	64.0	57.5	0.142	58.5	55.7
Z-curve	59.0	60.9	64.7	58.1	0.182	57.4	60.7
EIIP	58.4	62.3	65.5	57.7	0.171	57.4	59.6
RFF	63.4	66.4	66.6	64.9	0.271	68.3	58.5
Pse-in-One2.0	62.3	65.3	65.3	62.9	0.246	63.7	60.9
iLearn	60.7	62.9	64.1	59.9	0.215	59.3	62.0
SubFeat	59.6	63.0	67.0	58.9	0.195	57.4	61.7
Proposed features	**79.4**	**84.9**	**86.0**	**78.7**	**0.590**	**76.8**	**82.1**

**LncRNA dataset** Similarly for the lncRNA dataset, also the proposed features based on the PyFeat with AB achieve promising results compared with other techniques with the seven performance measures. As shown in Table  7, for 10-fold cross-validation, the proposed features achieved the following: ACC equals 77.8%, AUC equals 84.1%, AUPR equals 84.5%, F1-score equals 77.4%, MCC equals 0.560, SEN equals 77.3%, and SPC equals 78.3%. Also, after the proposed features, the features based on the RFF method achieve better results than the remaining methods: five representations with Fourier transform, Pse-in-one2.0, iLearn, and SubFeat.

**Figure 3:**
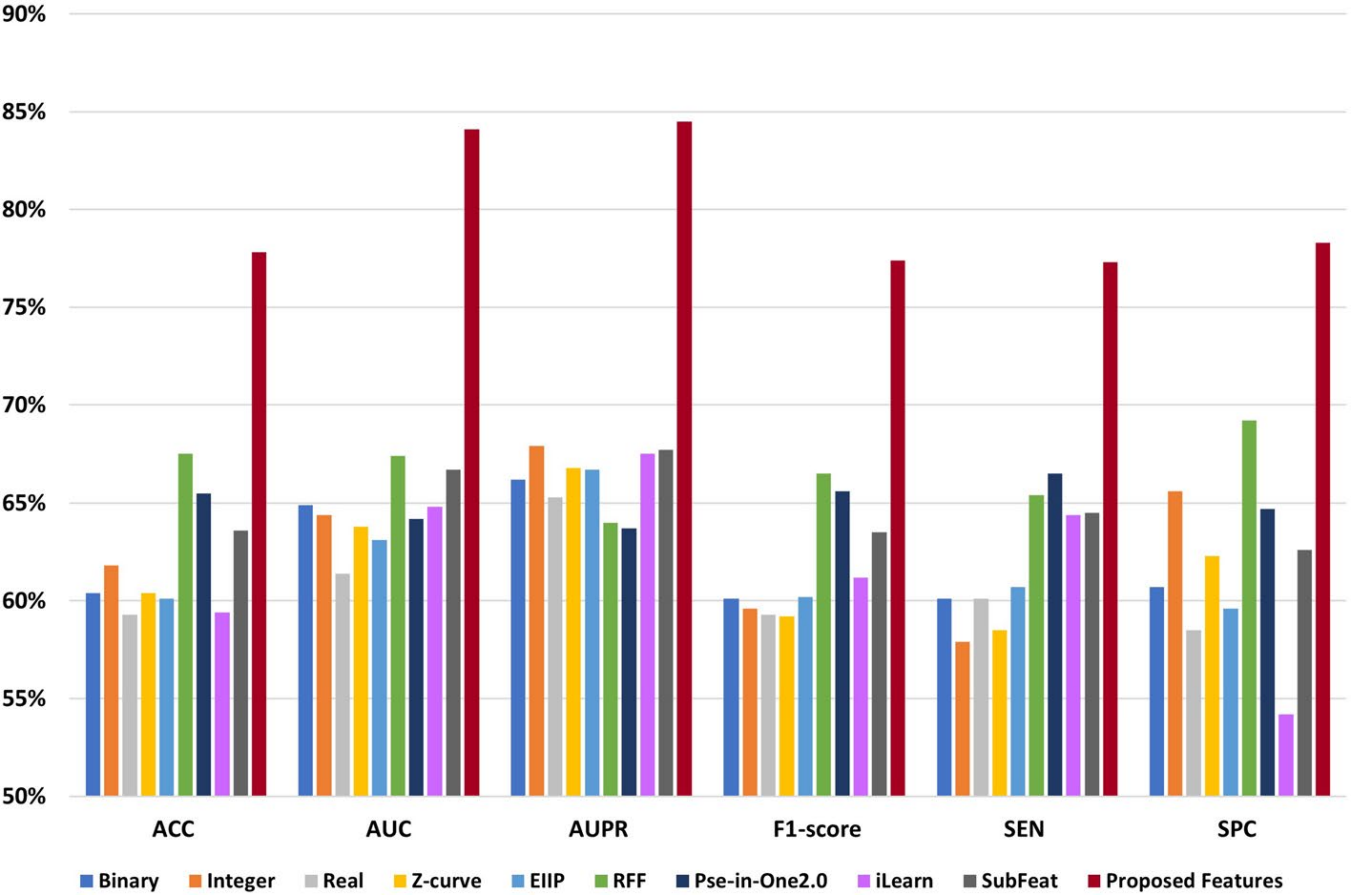
The performance evaluation of the features based on PyFeat with AB compared with other techniques: five numerical representations, RFF, Pse-in-One2.0, iLearn, and SubFeat based on the lncRNA dataset.

Meanwhile, the features based on the real method give the worst results compared with other methods. Figure 3 represents the comparison chart among performance measures of the features based on PyFeat with AB and other methods on the lncRNA dataset.

**Table 7:** The performance evaluation of the proposed features based on PyFeat with AB compared with other techniques: five numerical representations, RFF, Pse-in-One2.0, iLearn, and SubFeat with 10-fold cross-validation based on the lncRNA dataset. Significant values are in bold.

Metric	ACC (%)	AUC (%)	AUPR (%)	F1-score (%)	MCC	SEN (%)	SPC (%)
Binary	60.4	64.9	66.2	60.1	0.209	60.1	60.7
Integer	61.8	64.4	67.9	59.6	0.237	57.9	65.6
Real	59.3	61.4	65.3	59.3	0.190	60.1	58.5
Z-curve	60.4	63.8	66.8	59.2	0.214	58.5	62.3
EIIP	60.1	63.1	66.7	60.2	0.206	60.7	59.6
RFF	67.5	67.4	64.0	66.5	0355	65.4	69.2
Pse-in-One2.0	65.5	64.2	63.7	65.6	0.316	66.5	64.7
iLearn	59.4	64.8	67.5	61.2	0.193	64.4	54.2
SubFeat	63.6	66.7	67.7	63.5	0.278	64.5	62.6
Proposed features	**77.8**	**84.1**	**84.5**	**77.4**	**0.560**	**77.3**	**78.3**

#### Classification algorithm comparison

After the feature extraction step, the most important features were extracted based on the PyFeat method and the AB feature selection technique. These selected features were fed to the GBDT technique to diagnose different positive or negative cases. To validate the performance of the GBDT, the proposed system based on the GBDT classifier is compared with state-of-the-art classification algorithms. We evaluated the results based on protein and lncRNA datasets using seven performance measures with 4-fold and 10-fold cross-validation techniques to validate these datasets and overcome the overfitting limitations. In our experiments, we compared The GBDT with eight state-of-the-art classifiers: Logistic regression (LR)^58^, Decision tree (DT)^59^, Naive Bayes (NB)^60^, bagging^61^, RF^62^, AB^63^, SVM^64^, and linear discriminant analysis (LDA)^65^. The summary of the results in terms of ACC, AUC, AUPR , F1-Score, MCC, SEN, and SPC is given in Tables  8 and 9 based on protein and lncRNA datasets, respectively.

**Table 8:** The performance evaluation of the proposed system based on the GBDT compared with state-of-the-art classifiers using 4-fold and 10-fold cross-validation techniques on the protein dataset. Significant values are in bold.

Metric	K-fold	ACC (%)	AUC (%)	AUPR (%)	F1-score (%)	MCC	SEN (%)	SPC (%)
LR	4	65.7	71.7	68.7	64.5	0.316	63.5	67.9
	10	66.8	72.1	70.9	65.8	0.340	65.2	68.5
DT	4	62.4	62.5	57.7	61.8	0.250	61.3	63.6
	10	61.4	61.4	56.9	61.9	0.230	63.5	59.2
NB	4	48.5	47.6	47.4	17.8	− 0.097	22.1	74.5
	10	46.6	47.2	49.7	6.93	− 0.139	8.8	83.7
Bagging	4	66.6	72.3	69.4	61.8	0.340	54.7	78.3
	10	68.8	75.3	73.4	66.4	0.381	63.0	74.5
RF	4	75.3	83.8	83.4	74.2	0.508	71.8	**78.8**
	10	77.2	**84.9**	**86.2**	75.3	0.554	70.7	**83.7**
AB	4	72.3	80.7	78.2	72.6	0.449	74.0	70.7
	10	74.2	81.7	82.5	73.5	0.501	71.8	76.3
SVM	4	68.8	74.9	74.0	67.0	0.378	64.1	73.4
	10	68.8	75.4	75.6	67.8	0.378	66.9	70.7
LDA	4	59.5	60.5	58.2	58.6	0.190	57.5	61.4
	10	60.2	62.0	60.2	60.7	0.207	63.0	57.6
GBDT	4	**77.0**	**84.7**	**84.3**	**77.4**	**0.542**	**79.0**	75.0
	10	**79.4**	**84.9**	86.0	**78.7**	**0.590**	**76.8**	82.1

**Table 9:** The performance evaluation of the proposed system based on the GBDT compared with state-of-the-art classifiers using 4-fold and 10-fold cross-validation techniques based on the lncRNA dataset. Significant values are in bold.

Metric	K-fold	ACC (%)	AUC (%)	AUPR (%)	F1-score (%)	MCC	SEN (%)	SPC (%)
LR	4	62.5	70.3	70.8	62.0	0.254	62.3	62.6
	10	66.0	71.6	71.1	65.1	0.322	64.1	67.9
DT	4	62.0	61.9	57.3	60.5	0.246	61.3	62.6
	10	57.0	57.0	54.3	57.1	0.141	59.7	54.3
NB	4	54.9	55.0	52.6	66.9	0.164	91.5	18.7
	10	48.2	47.5	51.7	8.6	− 0.075	10.0	85.9
Bagging	4	71.4	75.7	73.2	68.7	0.433	64.2	78.5
	10	65.2	72.0	71.0	62.8	0.310	59.1	71.2
RF	4	71.8	80.0	82.6	70.2	0.442	68.9	74.8
	10	74.2	82.0	83.0	73.0	0.487	70.7	77.7
AB	4	73.7	79.4	75.4	74.1	0.478	75.5	72.0
	10	72.3	79.9	79.8	71.6	0.541	70.2	74.5
SVM	4	70.5	77.0	74.9	71.4	0.416	75.5	65.4
	10	67.67	74.5	74.0	66.4	0.355	65.2	70.1
LDA	4	55.9	60.2	57.5	55.4	0.120	56.6	55.1
	10	61.1	62.3	58.9	60.5	0.226	61.9	60.3
GBDT	4	**75.5**	**84.8**	**86.2**	**74.8**	**0.519**	**72.6**	**78.5**
	10	**77.8**	**84.1**	**84.5**	**77.4**	**0.560**	**77.3**	**78.3**

**Protein dataset** Table  8 shows the performance evaluation of the proposed prediction system based on the GBDT classifier compared with state-of-the-art classification algorithms. This table shows the best values achieved in this experiment in bold-faced fonts. For 4-fold cross-validation, the GBDT achieved ACC of 77.0%, AUC equals 84.7%, AUPR equals 84.3%, F1-score equals 77.4%, MCC equals 0.542, SEN equals 79.0%, and SPC equals 75.0%. For 10 fold cross-validation, the GBDT achieved ACC of 79.4%, AUC equals 84.9%, AUPR equals 86.0%, F1-score equals 78.7%, MCC equals 0.590, SEN equals 76.8%, and SPC equals 82.1%. The GBDT achieves promising results compared with other classifiers with 4-fold and 10-fold cross-validation based on the protein dataset. After the GBDT, the FR classifier shows better results than the remaining algorithms.

**Figure 4:**
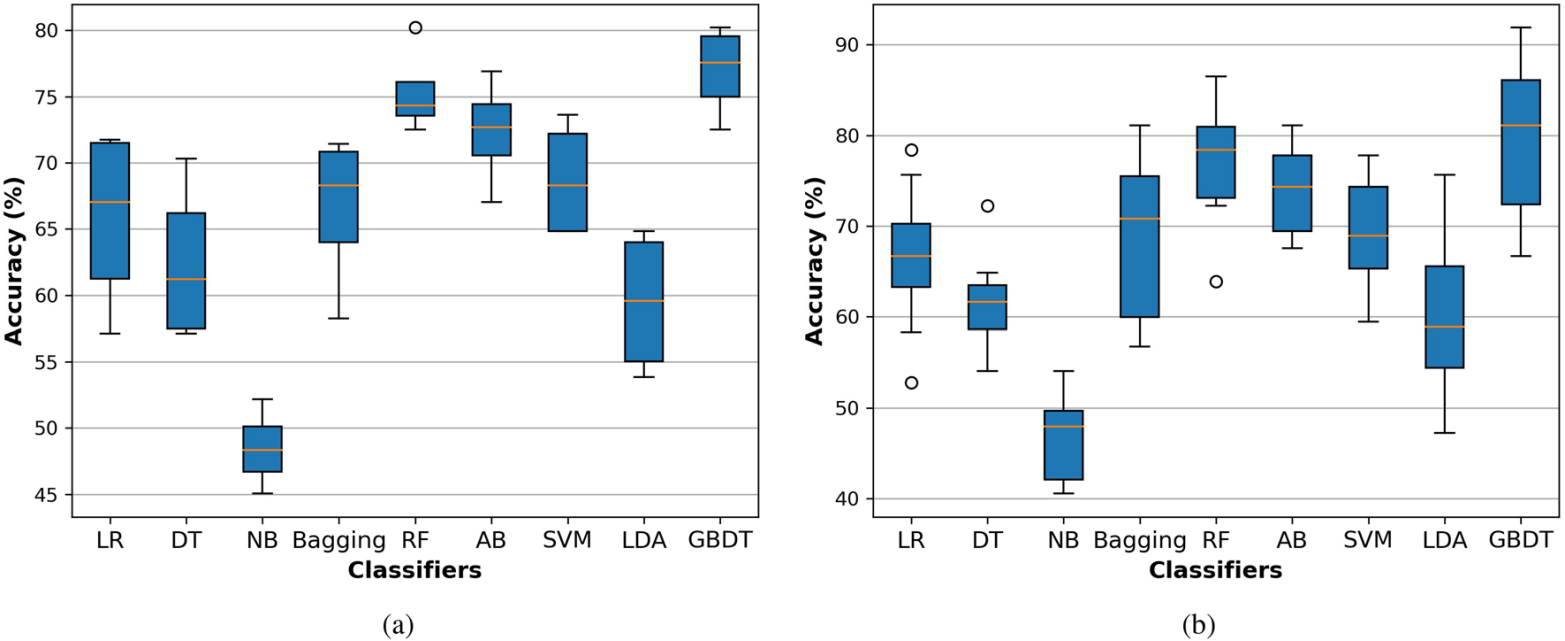
The AUC for the proposed system based on the GBDT compared with state-of-the-art classifiers on the protein dataset with (**a**) 4-fold and (**b**) 10-fold cross-validation techniques.

**Figure 5:**
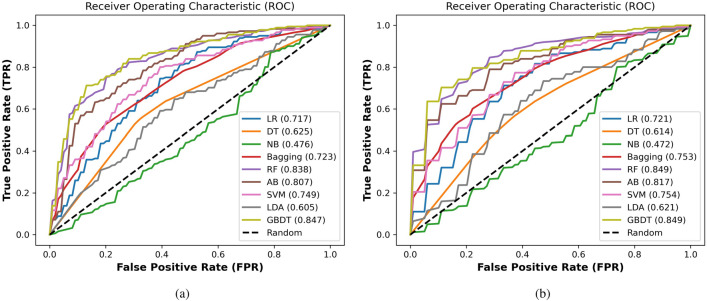
The accuracy box for the proposed system based on the GBDT compared with state-of-the-art classifiers on the protein dataset with (**a**) 4-fold and (**b**) 10-fold cross-validation techniques.

Meanwhile, the NB classifier performs as the worst classifier compared with other classification algorithms. The box plot of the accuracy of different classifiers is drawn with 4-fold and 10-fold cross-validation based on the protein dataset as shown in Fig. 4. From these plots based on the error bars, it is also proof that GBDT is better than other classification algorithms. We also provide the AUC for all classifiers with 4-fold and 10-fold cross-validation based on the protein dataset as shown in Fig. 5. Based on the area under the ROC, increasing in this area plays a role in improving the system accuracy for diagnosing the different test cases. The GBDT achieved promising results compared with other classifiers.

**LncRNA dataset** Similarly for the lncRNA dataset, The GBDT achieved promising results compared with state-of-the-art classifiers with 4-fold and 10 fold cross-validation. As shown in Table 9, for 4-fold cross-validation, the GBDT achieved ACC of 75.5%, AUC equals 84.8%, AUPR equals 86.2%, F1-score equals 74.8%, MCC equals 0.519, SEN equals 72.6%, and SPC equals 78.5%. For 10-fold cross-validation, the GBDT achieved ACC of 77.8%, AUC equals 84.1%, AUPR equals 84.5%, F1-score equals 77.4%, MCC equals 0.560, SEN equals 77.3%, and SPC equals 78.3%, as shown in Table 9. After the GBDT, the RF and AB classifiers show better results than the remaining algorithm.

**Figure 6:**
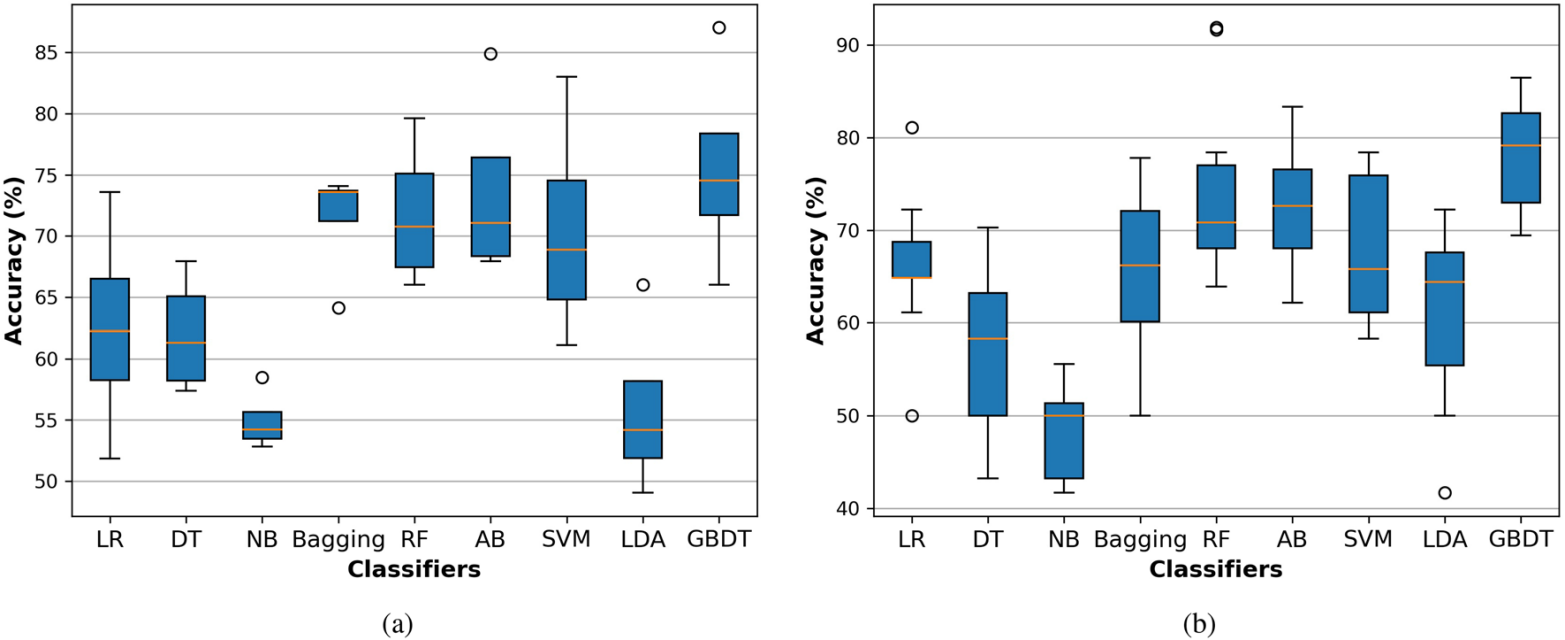
The accuracy box for the proposed system based on the GBDT compared with state-of-the-art classifiers on the lncRNA dataset with (**a**) 4-fold and (**b**) 10-fold cross-validation techniques.

**Figure 7:**
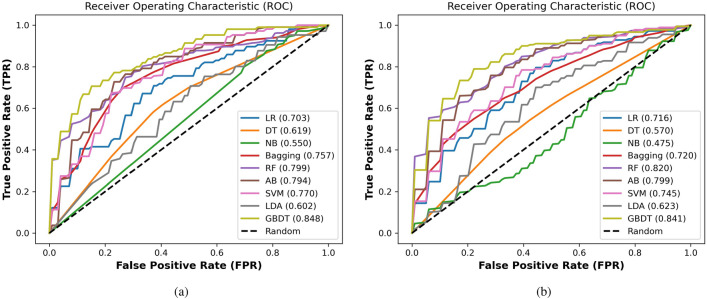
The AUC for the proposed system based on the GBDT compared with state-of-the-art classifiers on the lncRNA dataset with (**a**) 4-fold and (**b**) 10-fold cross-validation techniques.

Meanwhile, the NB classifier performs as the worst classifiers compared with other algorithms. We represent the box plot of accuracy of different classifiers with 4-fold and 10-fold cross-validation based on the lncRNA dataset as shown in Fig. 6. We also provide the AUC for all classifiers with 4-fold and 10-fold cross-validation based on the protein dataset as shown in Fig. 7. As shown in these Figs. 6 and 7, the GBDT shows promising results compared with state-of-the-art classifiers. Based on Tables 8 and 9, the 4-fold and 10-fold cross-validation techniques represented results that are very close to each other based on the proteins and lncRNA datasets. It is also evidence of the proposed system’s precision.

#### Comparison with other prediction systems

To validate the performance of the proposed system based on the PyFeat method with the AB feature selection technique, and the GBDT classification algorithm. First, we compared the performance of the proposed system with state-of-the-art systems: Bonidia et al.^34^, Nosrati et al.^66^, SUN et al.^67^, and Haque et al.^43^. Note that all these systems was built based on FASTA datasets and we reproduced their systems with our protein and lncRNA datasets.

We compare these system with our proposed system these systems with seven performance measures using 10-fold cross-validation technique. The summary of the results in terms of ACC, AUC, AUPR , F1-Score, MCC, SEN, SPC, classification algorithm, and feature selection method is given in Tables 10 and  11 based on the protein and lncRNA datasets respectively. The proposed system based on the PyFeat method with the AB technique achieves promising results compared with these systems with the seven performance measures using 10-fold cross-validation technique on protein and lncRNA datasets.

**Protein dataset** Table 10 shows the comparison of the proposed system with state-of-the-art systems based on the protein dataset. This comparison based on performance evaluation with 10-fold cross-validation technique, classification algorithm, and feature selection technique. The proposed system based on the PyFeat method with the AB feature selection technique, and the GBDT classification algorithm achieves promising results compared with other systems in the seven performance metrics. After the proposed system, Bonidia et al.^34^ system based on Z-curve method for feature extraction, RF classification algorithm, and without feature selection technique achieves better results than the remaining systems.

**Figure 8:**
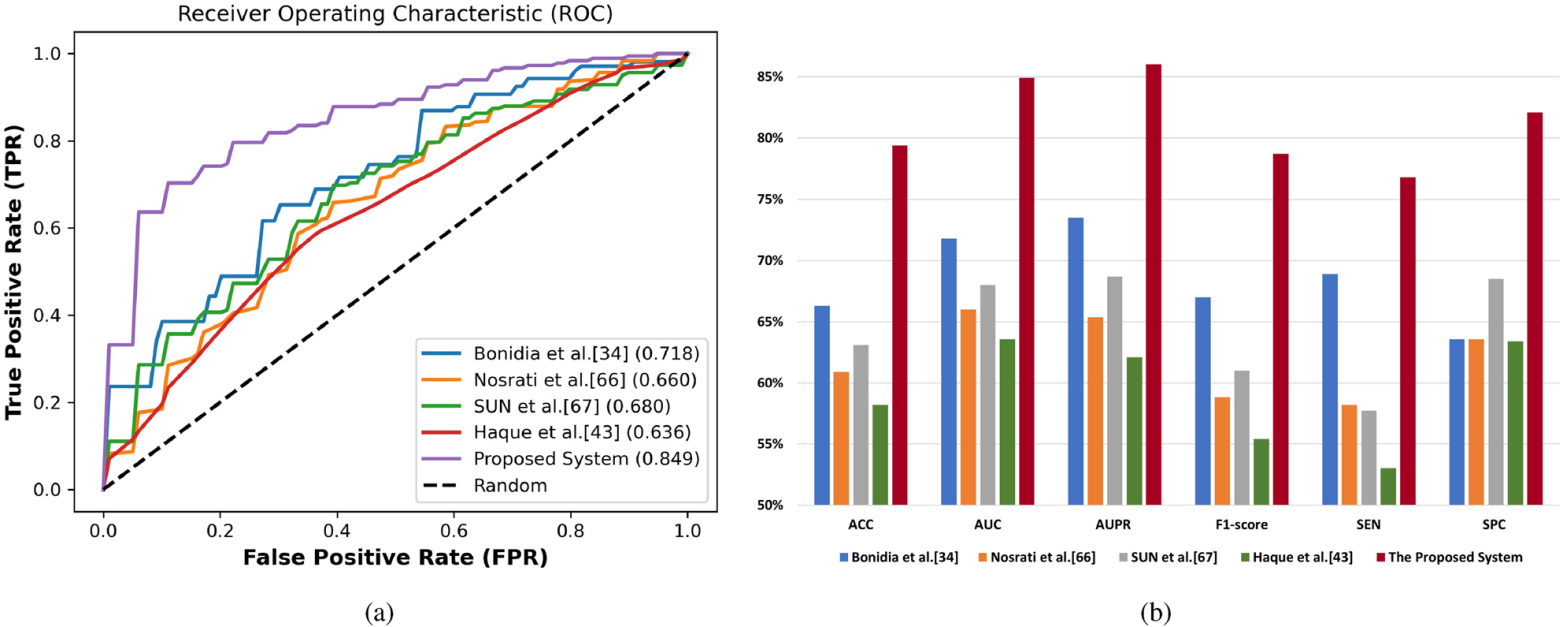
The comparison of the proposed system compared with the state-of-art systems based on the protein dataset. (**a**) AUC under ROC Curve. (**b**) Performance evaluation.

Meanwhile, Haque et al.^43^ system based on SubFeat technique for feature extraction and ensemble classifiers (SVM, SVM, SVM) is considered the worst compared with other systems. We also plot the ROC curve for our proposed system and other systems based on the protein dataset as shown in Fig. 8a. From these curves, it is also evident that our proposed system is better than others systems, as the increasing in this area will improving the prediction model. Figure 8b summarizes the performance results of the proposed system and other systems based on the protein dataset.

**Table 10:** The performance comparison, classification methods, and feature selection methods used in state-of-art systems compared with the proposed system based on the protein dataset. Significant values are in bold.

System	ACC (%)	AUC (%)	AUPR (%)	F1-score (%)	MCC	SEN (%)	SPC (%)	Classification method	Feature selection method
Bonidia et al.^34^	66.3	71.8	73.5	67.0	0.331	68.9	63.6	RF	None
Nosrati et al.^66^	60.9	66.0	65.4	58.8	0.219	58.2	63.6	RF	None
SUN et al.^67^	63.1	68.0	68.7	61.0	0.266	57.7	68.5	SVM	F-score, Greedy Algorithm
Haque et al.^43^	58.2	63.6	62.1	55.4	0.166	53.0	63.4	SVM, SVM, SVM	None
Proposed system	**79.4**	**84.9**	**86.0**	**78.7**	**0.590**	**76.8**	**82.1**	**GBDT**	**AdaBoost**

**Figure 9:**
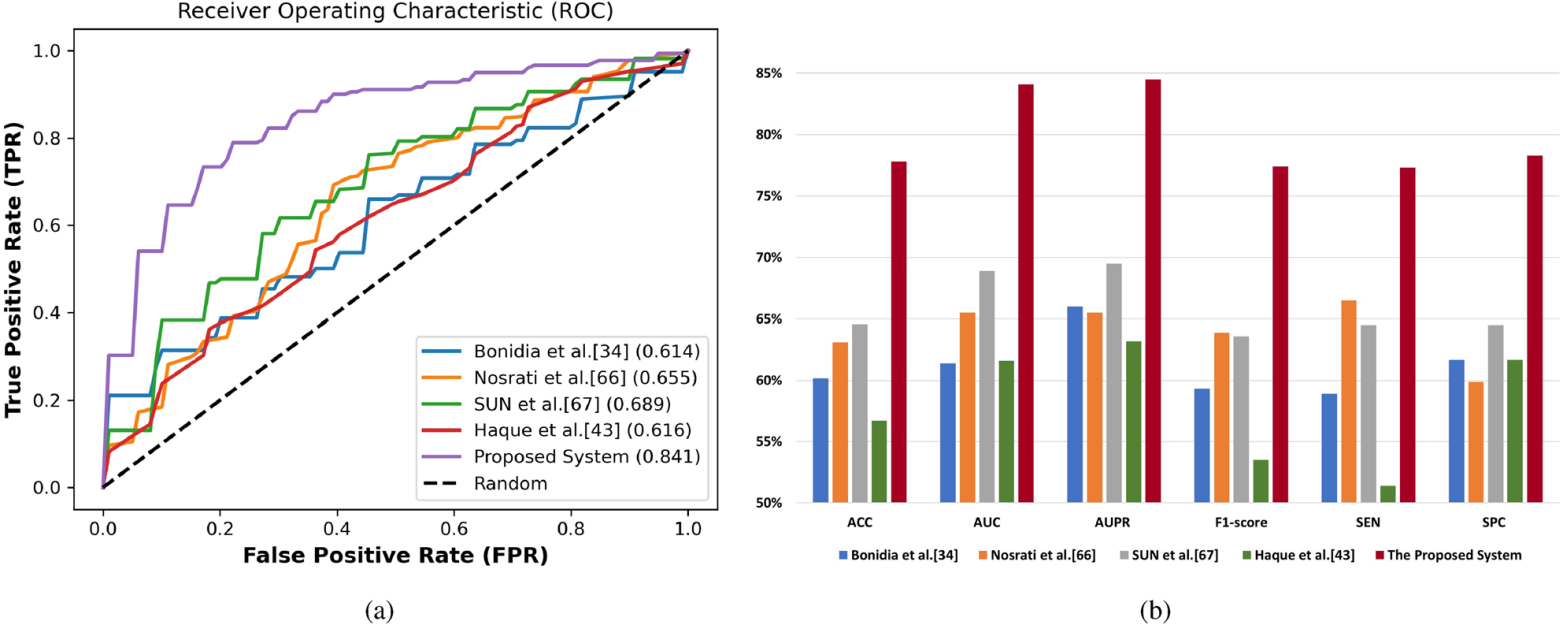
The comparison of the proposed system compared with the state-of-art systems based on the lncRNA dataset. (**a**) AUC under ROC Curve. (**b**) Performance evaluation.

**LncRNA dataset** Similarly for the lncRNA dataset, the prediction system achieve promising results compared with other systems, as shown in Table 11. After our proposed system, SUN et al.^67^ system based on iLearn technique for feature extraction, SVM classification algorithm, and F-score and greedy algorithm feature selection techniques^68^, shows better results than the remaining systems. Meanwhile, also Haque et al.^43^ system based on SubFeat for feature extraction, ensemble classifiers (SVM, SVM, SVM), and without feature selection technique, is considered the worst compared with other systems. Also ROC curve proved this point as shown in Fig. 9a. Figure 9b summarizes the performance results of the proposed system and other systems based on the lncRNA dataset. Based on Tables 10 and 11, its are also evidence that the proposed prediction system is better than state-of-the-art systems based on the proteins and lncRNA datasets.

**Table 11:** The performance comparison, classification methods, and feature selection methods used in state-of-art systems compared with the proposed system based on the lncRNA dataset. Significant values are in bold.

System	ACC (%)	AUC (%)	AUPR (%)	F1-score (%)	MCC	SEN (%)	SPC (%)	Classification method	Feature selection method
Bonidia et al.^34^	60.2	61.4	66.0	59.3	0.210	58.9	61.7	RF	None
Nosrati et al.^66^	63.1	65.5	65.5	63.9	0.266	66.5	59.9	RF	None
SUN et al.^67^	64.6	68.9	69.5	63.6	0.299	64.5	64.5	SVM	F-score, Greedy Algorithm
Haque et al.^43^	56.7	61.6	63.2	53.5	0.418	51.4	61.7	SVM,SVM,SVM	None
Proposed system	**77.8**	**84.1**	**84.5**	**77.4**	**0.560**	**77.3**	**78.3**	**GBDT**	**AdaBoost**

**Figure 10:**
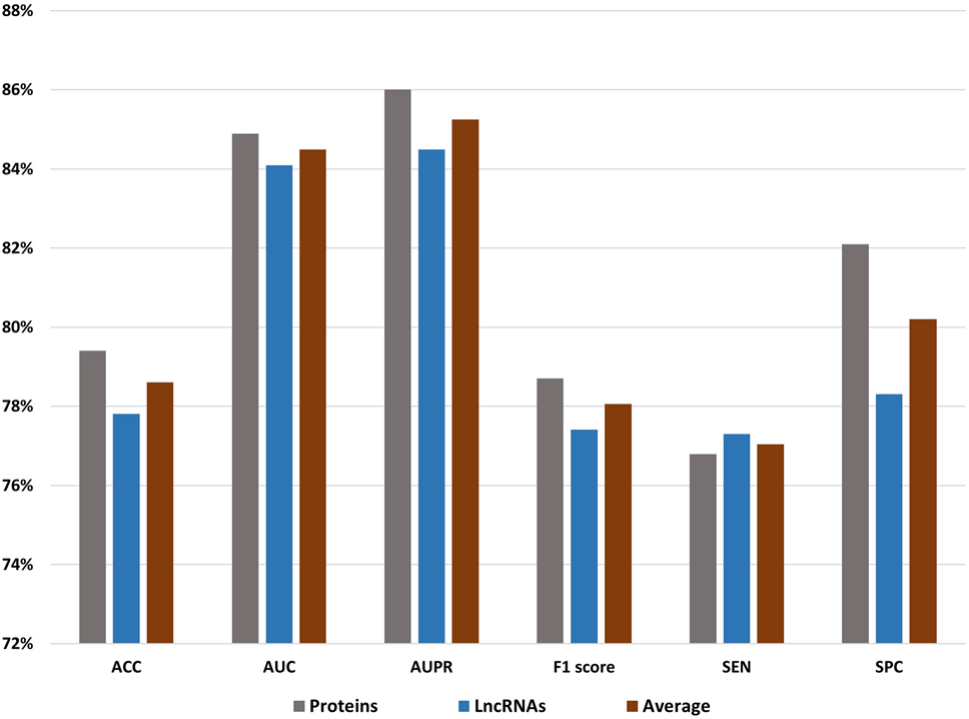
The average performance evaluation of the proposed prediction system based on protein and lncRNA datasets.

Secondly, based on the results of the proposed system on two datasets in Tables 8 and  9, We compute the average performance of our proposed system with 10-fold cross-validation technique based on protein and lncRNA datasets. In Table 12, We noticed that the protein dataset achieved ACC of 79.4%, AUC equals 84.9%, AUPR equals 86.0%, F1-score equals 78.7%, MCC equals 0.590, SEN equals 76.8%, and SPC equals 82.1%. On lncRNA dataset achieved ACC of 77.8%, AUC equals 84.1%, AUPR equals 84.5%, F1-score equals 77.4%, MCC equals 0.560, SEN equals 77.3%, and SPC equals 78.3%. So that, the average evaluation for our proposed system as follows: ACC equals 78.6%, AUC equals 84.5%, AUPR equals 85.3%, F1-score equals 78.3%, MCC equals 0.575, SEN equals 77.1%, and SPC equals 80.2%. Figure 10 summarizes the performance results based on the proteins, lncRNAs, and the average results of the proposed prediction system as demonstrated in Table 12.

**Table 12:** Average performance of the proposed prediction system based on protein and lncRNA datasets.

Datasets	ACC (%)	AUC (%)	AUPR (%)	F1-score (%)	MCC	SEN (%)	SPC (%)
Proteins	79.4	84.9	86.0	78.7	0.590	76.8	82.1
LncRNAs	77.8	84.1	84.5	77.4	0.560	77.3	78.3
Average	78.6	84.5	85.3	78.1	0.575	77.1	80.2

**Figure 11:**
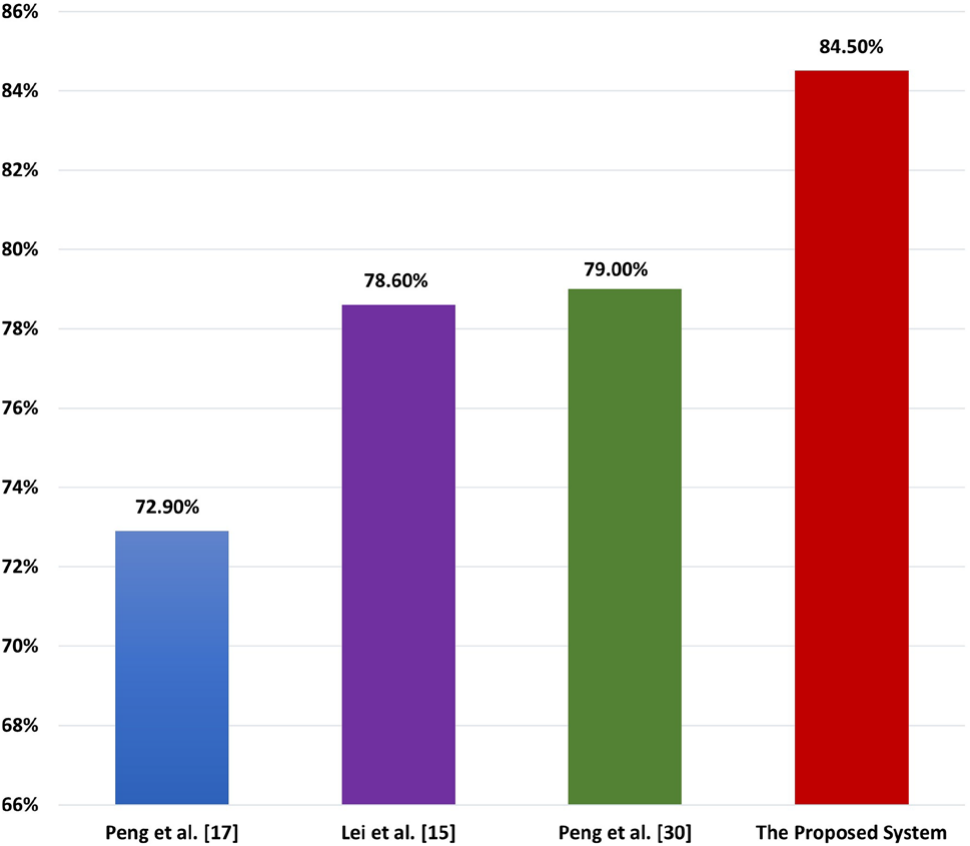
The comparison between our proposed system and some current studies based on AUC.

Finally, we compared the proposed system with state-of-the-art systems: Peng et al.^17^, Lei et al.^15^, and Peng et al.^30^. Note that, these studies applied their experiments for predicting genes related to PD and on the same dataset that we are using in the proposed prediction system. Note that, their results are taken as reported in their studies, and they evaluated their systems based on only the AUC performance metric. The AUC for Peng et al.^17^, Lei et al.^15^, and Peng et al.^30^ equals%72.9, 78.6%, and 79.0%, respectively. Meanwhile, the proposed system achieved 84.5% AUC as an AUC average based on the protein and lncRNA dataset results, as shown in Table 13. Based on Table 13, our system achieve promising results compared with state-of-the-art prediction system for PD. Figure 11 represents the comparison chart among the AUC of our proposed prediction system and other prediction systems.

**Table 13:** The comparison between our proposed system and some current systems based on AUC. Significant value is in bold.

	Peng et al.^17^	Lie et al.^15^	Peng et al.^30^	The proposed system
AUC (%)	72.9	78.6	79.0	**84.5**

## Discussion

PD is considered the most common movement disease and the second most common neurodegenerative disease after AD. Several cardinal signs are associated with PD: tremor, rigidity, bradykinesia, and postinstability. Thus, to avoid these symptoms, we need to diagnose the disease early . Identifying and predicting disease-related genes have biological significance in most biomedical studies, which aid in an early diagnosis and treatment of the disease. Consequently,, identifying genes related to PD is crucial to the disease’s diagnosis and treatment. The recent PD gene prediction studies utilized the proteins’ genes and discard lncRNA genes related to the PD. However, lncRNAs are essential in the metastasis and progression of various diseases. Consequently, we built our proposed prediction system for identifying protein and lncRNA genes related to PD.

In this study, we utilized two datasets for the protein and lncRNA genes, then we represented all genes as DNA FASTA sequences and removed the replicate sequences in FASTA files. To evaluate the proposed system, we used 4-fold and 10-fold cross-validation techniques. The most critical features are extracted using the PyFeat method with the AB as a feature selection technique. These features achieved the best results compared with extracted features from state-of-the-art feature extraction techniques: five numerical representations with Fourier transform, RFF, Pse-in-One2.0, iLearn, and SubFeat. The selected features are fed to the GBDT technique to diagnose different test cases and build our model to identify genes related to PD. Also, the GBDT is compared with the state-of-the-art classification algorithms. It is also proof that the proposed system with GBDT is better than other classification algorithms. To validate our proposed system based on the PyFeat with AB, and GBDT, it compared with state-of-the-art systems, which used FASTA sequences datasets in their studies: Bonidia et al.^34^, Nosrati et al.^66^, SUN et al.^67^, Haque et al.^43^. This comparison is also evidence that our proposed system achieves promising results compared with these state-of-the-art systems.

We evaluated the results using seven performance evaluation measures. For 10-fold cross-validation technique, the protein dataset achieved the following: ACC equals 79.4%, AUC equals 84.9%, AUPR equals 86.0%, F1-score equals 78.7%, MCC equals 0.590, SEN equals 76.8%, and SPC equals 82.1%. On the lncRNA dataset, ACC equals 77.8%, AUC equals 84.1%, AUPR equals 84.5%, F1-score equals 77.4%, MCC equals 0.560, SEN equals 77.3%, and SPC equals 78.3%. The average results based on the protein and lncRNA dataset are as follows: ACC equals 78.6%, AUC equals 84.5%, AUPR equals 85.3%, F1-score equals 78.3%, MCC equals 0.575, SEN equals 77.1%, and SPC equals 80.2%.

Also, the proposed prediction system is compared with some state-of-the-art studies: Peng et al.^17^, Lie et al.^15^, and Peng et al.^30^, that build their models for predicting genes related to PD and used the same datasets that we are using in our experiments. Peng et al.^17^ identified proteins related to PD using the N2A-SVM model with AUC equals %72.9 based on ClinVar dataset. Peng et al.^30^ identified protein genes related to disease with AUC equals 78.6% based on the SLN-SRW model on Clinvar, GO, DO, and OMIM datasets. Lie et al.^15^ predicted protein and lncRNA genes related to diseases with AUC equals 79.0% using InLPCH model on LncRNADisease, HPRD, and OMIM datasets. Based on the protein and lncRNA dataset results, the proposed system achieved AUC equals 84.5% as an AUC average. This comparison is also evidence that our proposed system achieves promising results compared with these systems. Meanwhile, the proposed prediction system is used to predict and identify protein and lncRNA genes related to PD compared with other systems that identified only protein genes.

Finally, we used the proposed prediction system to predict new protein and lncRNA genes related to PD, which are not found in the databases. These genes are ranked according to the probability predicted by the training model. Then, the top 10 protein and lncRNA genes are selected, and the literature review is used to verify these genes. For proteins, the 10 genes were extracted: PACRG, GIA5, TH, LRRK2, TNR, VCP, KCNJ2, SETX, APBB1, and DCTN1. Based on the literature review, we discovered that some of these genes had been reported to be associated with PD. PACRG, TH, LRRK2, TNR, and VCP are reported in^69–75^. Additionally, KCNJ2, APBB1, and DCTN1 genes are associated with neurodegenerative diseases^76–78^. The GJAS gene is related to a gene associated with PD^79^. Finally, the SETX gene is related to the tremor, which is considered a sign of PD^80^. For the LncRNAs, the 10 genes were extracted: PDZRN3, NEAT1, DAOA-AS1, TUG1, PPP3CB, DAPK1, H19, MAPT-AS1, MESTIT1, and PCA3. Based on the literature review, we discovered that some of these genes had been reported to be associated with PD. NEAT1, TUG1, DAPK1, H19, MATP-AS1, and PCA3 genes were reported in^81–86^. Additionally, PDZRN3 and PPP3CB genes are associated with neurodegenerative diseases, as reported in^87,88^. The MESTIT1 gene is associated with a cognitive disease, as reported in^89^. Finally, the DAOA-AS1 gene is extracted for bipolar disorder as reported in^90^.

## Conclusion

We developed a novel prediction system for identifying genes related to PD that involve proteins and lncRNAs. We used two public databases: ClinVar for proteins and LncRNADisease V2.0 for lncRNAs. The proposed prediction system comprises four steps. First, we represented the genes as DNA FASTA sequences from the UCSC genome browser and removed the replicates sequences from the FASTA file as a preprocessing step. Second, we extracted the most significant features of the DNA FASTA sequences using the PyFeat method with the AB as a feature selection technique. Then, the selected features were fed to the GBDT technique to diagnose different test cases. Finally, seven performance metrics are used to evaluate the results of the proposed system. In the future, we aim to identify gene changes concerning the different grades of PD. Meanwhile, we aim to apply our proposed prediction system to identify and predict other diseases with related genes.

## Data Availability

The datasets used during the current study available in the ClinVar (https://www.ncbi.nlm.nih.gov/clinvar/) for proteins dataset, and in the LncRNA v2.0 (http://www.rnanut.net/lncrnadisease/) for the lncRNAs dataset. Also, the datasets used during the current study available from the corresponding author on responsible request.
